# Predicting mosquito flight behavior using Bayesian dynamical systems learning

**DOI:** 10.1126/sciadv.adz7063

**Published:** 2026-03-18

**Authors:** Christopher Zuo, Chenyi Fei, Alexander E. Cohen, Soohwan Kim, Ring T. Cardé, Jörn Dunkel, David L. Hu

**Affiliations:** ^1^ Woodruff School of Mechanical Engineering, Georgia Institute of Technology, Atlanta, GA 30332, USA.; ^2^ Department of Mathematics, Massachusetts Institute of Technology, Cambridge, MA 02139, USA.; ^3^ Department of Entomology, University of California, Riverside, CA 92521, USA.; ^4^ School of Biological Sciences, Georgia Institute of Technology, Atlanta, GA 30332, USA.

## Abstract

Mosquito-borne diseases cause several hundred thousand deaths worldwide every year. Deciphering mosquito host-seeking behavior is essential to prevent disease transmission through mosquito capture and surveillance. Despite recent substantial progress, we still lack a comprehensive quantitative understanding of how visual and other sensory cues guide mosquitoes to their targets. Here, we combined three-dimensional infrared tracking of *Aedes aegypti* mosquitoes with Bayesian dynamical systems inference to learn a quantitative biophysical model of mosquito host-seeking behavior. Trained on more than 20 million data points, each corresponding to an instantaneous position and velocity in mosquito free-flight trajectories recorded in the presence of visual and carbon dioxide cues, the model accurately predicts how mosquitoes respond to human targets. Our results provide a quantitative foundation for optimizing mosquito capture and control strategies, a key step toward mitigating the impact of mosquito-borne diseases.

## Teaser

Millions of mosquito trajectories are used to predict how sensory cues guide mosquito fight patterns around humans.

## INTRODUCTION

Mosquitoes are often called the world’s most dangerous animals as they transmit diseases such as malaria, dengue fever, yellow fever, and Zika, which collectively cause more than 770,000 deaths annually (*1*, *2*). Among the 3500 mosquito species, ~100 species have evolved to be anthropophilic, meaning they preferentially target human hosts (*3*, *4*). *Aedes aegypti* is one such anthropophilic mosquito species, and females use a suite of cues to locate a human host (*5*–*13*). These cues have mostly been tested in wind tunnels, where airflow carries chemical signals like odor molecules and carbon dioxide downwind (*6*, *14*, *15*). Up to distances of 10 m downwind, emitted carbon dioxide triggers mosquito flight toward the host, lowers their threshold of detecting skin odors (*16*, *17*), and enhances their navigation toward visual targets (*18*). In addition to chemical cues, visual stimuli can guide mosquito flight to within meters of the host (*6*). Once in direct contact with the host, cues like skin odor, heat, and humidity (*19*) aid the mosquito in landing and choosing a spot to probe for capillaries (*20*). Despite decades of study, how mosquitoes integrate these cues to locate hosts remains poorly understood. Due to our inability to predict mosquito flight behavior, commonly used devices such as suction traps are only 10 to 50% effective in capturing incoming mosquitoes (*15*, *21*, *22*). An improved understanding of mosquito flight and host-seeking behaviors can help inform the design of more efficient intervention strategies for insect-borne diseases as well as the development of mosquito-resistant infrastructure in private and public spaces (*23*).

Previous investigations of mosquito host-seeking behavior were typically limited to small numbers of tethered mosquitoes or mosquitoes flying in wind tunnels (*6*, *15*, *22*, *24*). These studies often report statistics of various manually annotated metrics, such as the number and positions of mosquito landings, rather than providing continuous flight information (*25*–*29*). However, flight trajectories are crucial for understanding mosquito host-seeking behavior, as mosquitoes use the time-integrated response of their sensory information to make a host-seeking decision (*11*, *30*). Moreover, mosquito eyes limit the resolution and distance at which they can distinguish visual stimuli (*31*–*33*). Capturing the three-dimensional (3D) sensory information and the 3D flight trajectories necessitates a data-driven approach due to the size and dimensionality of the resulting datasets. The data-driven identification of interpretable biophysical models of mosquito flight not only will substantially enhance our understanding of mosquito host-seeking behavior under realistic environmental conditions but also can provide a proof-of-concept framework for model identification in other swarming and nonswarming species, such as bees, ants, starlings, and humans (*34*–*39*). Here, we introduce such a framework by combining 3D tracking experiments with Bayesian dynamical systems inference (*40*–*48*). Throughout, we use “inference” and “learning” interchangeably in the statistical sense to mean identifying a model and its parameters from experimental data (*49*).

To establish a foundational database of various mosquito flight behaviors, we perform 3D tracking experiments on female *Ae. aegypti* mosquitoes interacting with visual and CO_2_ cues, both individually and in combination (*50*–*52*). Because *Aedes* thrives in urban areas, our experiments are performed in relatively windless conditions, as typical of human dwellings with closed windows. Across 20 experiments, we record more than 53 million data points and more than 400 thousand mosquito trajectories, exceeding most previous attempts to quantitatively measure mosquito behavior (tables S1 and S2) (*8*, *21*, *27*–*29*, *53*–*57*). We then apply sparse Bayesian dynamical systems inference (*40*–*48*) to learn quantitative models of mosquito behavior directly from the mosquito flight trajectories. This data-driven method produces a predictive dynamical model from continuous flight data alone yet allows us to compute various behavioral traits of interest.

## RESULTS

### Tracking of 3D mosquito trajectories

**Fig. 1.**
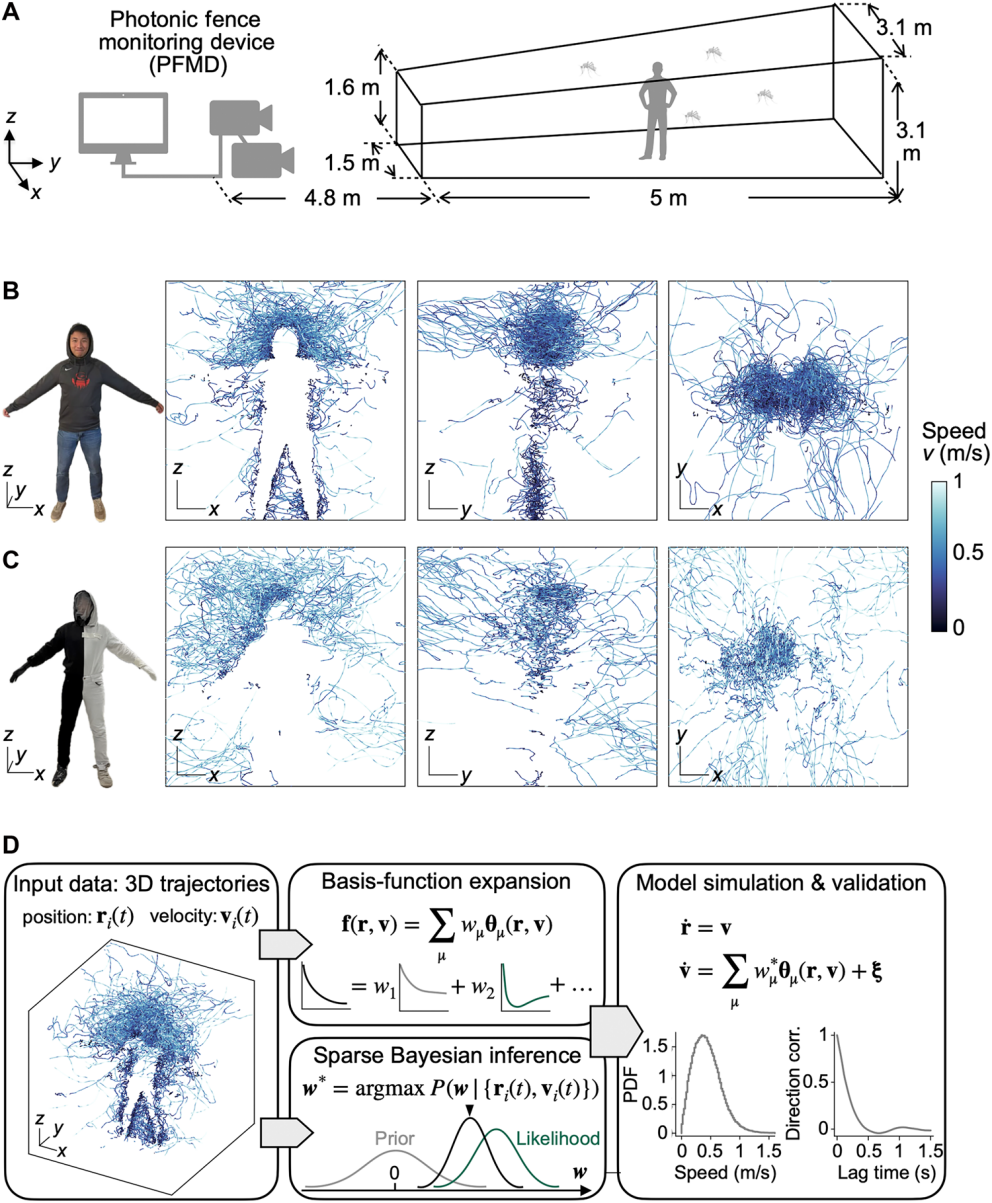
3D tracking of individual mosquitoes enables Bayesian dynamical systems learning of mosquito flight behaviors. (**A**) Design of the experimental setup. (**B**) Left: A human subject wearing dark clothing . The remaining panels show 2D projections of 3D mosquito trajectories around the subject shown on the left, colored by flight speed. Photo subject: C.Z. Photographer: D.L.H., Georgia Institute of Technology. (**C**) Left: A human subject wearing a half-black, half-white outfit. The remaining panels show 2D projections of 3D mosquito trajectories around the subject shown on the left, colored by flight speed. Photo subject: C.Z. Photographer: D.L.H., Georgia Institute of Technology. (**D**) Learning dynamical models for mosquito flight behaviors. Left: Inputs are 3D trajectories of mosquito positions r(t) and velocities v(t). Middle: The unknown behavioral force f is decomposed on a set of basis functions $\theta_{\mu }$, where the decomposition coefficients $w_{\mu }$ are learned from input data using sparse Bayesian inference. Right: The learned dynamical model can be simulated to generate synthetic time-series data for validation against input data, comparing them based on ensemble statistics such as speed probability density function (PDF) and directional correlation function, which are not used in model learning. Scale bars labeled with *x*, *y*, and *z*: 25 cm. For visual clarity, 50% of trajectories are shown.

We conducted the experiments at 28°C and 45% humidity inside a trapezoidal mesh enclosure of 5-m depth (Fig. 1A). Facing the enclosure was the photonic fence monitoring device (PFMD), which is a set of dual infrared cameras surrounded by infrared 850-nm light-emitting diodes (LEDs). The LEDs illuminate a scene against a retroreflective background, allowing for the dual cameras to obtain stereoscopic images of insect positions in space at 0.01-s resolution. Mosquitoes were released at the front of the chamber, and various targets (human, expanded polystyrene spheres, and carbon dioxide sources) were placed 8 m away from the camera.

As an homage to A. W. A. Brown’s 1951 experiments with dark-clothed manikins (*58*), we first applied the PFMD to image mosquito trajectories around a human subject wearing dark clothing (Fig. 1, A and B). In this experiment, our system tracked 50 mosquitoes for 20 min at a time resolution of 0.01 s and recorded 22 thousand positional trajectories of mosquitoes in 3D. A trajectory represents a segment of the full flight path of one mosquito, with an average duration of 0.9 s. While we cannot directly measure landing positions of mosquitoes because trajectories are lost when a mosquito stops moving or flies within the silhouette of the human subject, our experiment shows that *Ae. aegypti* mosquitoes primarily target the human head (Fig. 1B and movie S1), consistent with previous studies of landing location (*25*). As shown by the darker colors of the trajectories in Fig. 1B, mosquitoes decelerate near the human head and body, suggesting preparation for landing. The complex 3D tracks reflect the mosquitoes’ response to the multiple cues detected.

To demonstrate the importance of visual cues in windless situations, we tracked mosquitoes around a human subject wearing a Janus outfit with the left side white and the right side black. As can be seen from the front and top views, the trajectories are primarily concentrated on the black side, despite other cues such as carbon dioxide and odor remaining symmetric (Fig. 1C and movie S2). Because of the complexity of evaluating the cues around a human subject, we pivot from human experiments and proceed with experiments using simple objects that present individual cues. These controlled experiments allow for simple formulation of free-flight characteristics and a minimal model of *Ae. aegypti* in response to individual cues. Further below, we revisit the prediction of mosquito behavior around a human subject using our minimal model.

### Learning dynamical models for mosquito flight

To model mosquito flight behavior, we consider a stochastic Langevin dynamics

$$\frac{dv}{\mathrm{dt}}=f\left(r,v\right)+\xi$$ (1)

where f is a deterministic force (per mosquito body mass) that depends on the spatial position r of a mosquito and its flight velocity v and captures both the intrinsic active force of free flight, and the mosquito’s responses to environmental cues. The Gaussian white noise term ξ accounts for random fluctuations and satisfies 〈ξ(t)〉=0 and $\langle \xi \left(t\right)\xi \left(t^{\prime }\right)\rangle =\Delta I\delta \left(t-t^{\prime }\right)$, where I is the identity matrix and Δ characterizes the magnitude of noise. Throughout, we use light-face letters to denote scalar quantities and bold-face letters to denote vectors (overhat symbols indicate unit vectors). To learn a model of mosquito flight behavior from data, we approximate the unknown forces f(r,v) using a basis-function expansion (*48*, *59*–*61*) (Fig. 1D)

$$f\left(r,v\right)=\sum_{\mu }w_{\mu }\theta_{\mu }\left(r,v\right)$$ (2)

where $\theta_{\mu }$ represents an orthogonal vector basis and μ is a single index or a tuple of indices of the basis (see the Supplementary Materials). The coefficients $w_{\mu }$ encode all the information about mosquito behavior, with each environmental cue corresponding to a distinct set of $w_{\mu }$. This linear expansion in Eq. 2 simplifies the behavioral learning problem by reducing it to a linear regression problem.

To learn $w_{\mu }$ from data, we use a Bayesian approach to find the most probable coefficients $w_{\mu }^{*}$ that maximize the posterior probability $P\left(\{w_{\mu }\}|\{r_{i}\left(t\right),v_{i}\left(t\right)\}\right)$ of $w_{\mu }$ given the experimental trajectories, where i denotes the index of individual mosquito trajectories $\left\{r_{i}\left(t\right),v_{i}\left(t\right)\right\}$. In Bayesian statistics, the posterior is proportional to the product of two components (Fig. 1C): a likelihood function $P\left(\{r_{i}\left(t\right),v_{i}\left(t\right)\}|\{w_{\mu }\}\right)$ of the observed trajectories given the coefficients $\left\{w_{\mu }\right\}$ and a prior probability $P\left(\{w_{\mu }\}\right)$ that reflects our preference for the desired coefficients $\left\{w_{\mu }\right\}$. Assuming that each mosquito track is independent and neglecting mosquito-mosquito interactions, which we find to be minor (see fig. S1), Eq. 2 yields a multivariate Gaussian likelihood function (see the Supplementary Materials). To prevent overfitting to noisy data, we impose sparsity on $\left\{w_{\mu }\right\}$, favoring a parsimonious representation of smooth functions f(r,v) with few nonzero $w_{\mu }$. Following previous works on sparse Bayesian inference (*40*), we use a sparsity-promoting Gaussian prior $P\left(w_{\mu }\right)\propto \text{exp}\left(-\frac{w_{\mu }^{2}}{2\sigma_{\mu }^{2}}\right)$ where the variance hyperparameters $\sigma_{\mu }^{2}$ control the degree of sparsity in $\left\{w_{\mu }\right\}$. To estimate the coefficients $w_{\mu }^{*}$, the noise magnitude $\Delta^{*}$, and the hyperparameters $\sigma_{\mu }^{2}$, we use an expectation-maximization algorithm to iteratively maximize the posterior and refine model parameters (see the Supplementary Materials).

To explore a series of candidate models with varying degrees of sparsity, we apply sequential thresholding to $w_{\mu }^{*}$ by progressively eliminating coefficients with small absolute values (*62*–*64*). Although including more terms in the expansion in Eq. 2 can generally improve the fit to data, overly complex models may capture noise in the data rather than the true underlying forces f(r,v) and exhibit poor predictive power. Thus, to identify the model with optimal sparsity, we use the Bayesian information criterion (BIC) (*65*) to select the model that best explains the data with the least number of terms in Eq. 2 (see fig. S2). We validated our model identification pipeline on synthetic data of mosquito flight trajectories (figs. S3 and S4). Below, we apply this pipeline to experimental data to uncover dynamical models of mosquito flight behavior.

### *Ae. aegypti* exhibit two distinct modes of free flight

**Fig. 2.**
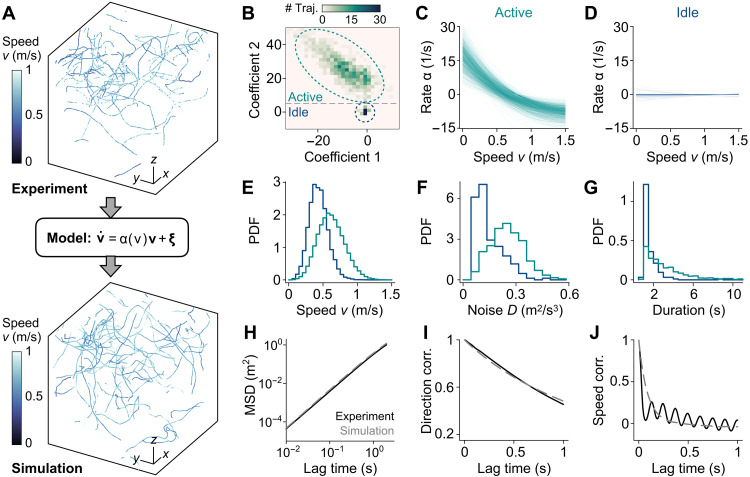
Free flying mosquitoes exhibit two distinct behaviors. (**A**) Representative mosquito trajectories from (top) experiments and (bottom) simulations of the learned model. Scale bars labeled with *x*, *y*, and *z*: 25 cm. For visual clarity, only 10% of total trajectories are shown. (**B**) 2D histogram of the two most relevant learned decomposition coefficients shows two groups of trajectories, an active group (green) and an idle group (blue). (**C** and **D**) The learned rates α at varying speed *v* for (C) the active group and (D) the idle group. Positive and negative α indicate acceleration and deceleration, respectively. (**E** to **G**) Probability distributions of (E) the measured flight speed, (F) the learned velocity diffusion coefficient, and (G) the duration of the trajectories for the two groups. (**H**) Mean squared displacement (MSD), (**I**) directional correlation, and (**J**) speed correlation are compared between experiments (black) and simulations of the learned model (gray). MSD shows ballistic flight at short lag times, transitioning to more diffusive movement around a lag time of 1 s. Directional correlation indicates straight flight over short intervals and turning over longer times, while speed correlation decreases toward zero, with experimental data showing small oscillations.

We first demonstrate our model learning and selection framework on experimental trajectories of free-flight mosquitoes in the absence of sensory stimuli (Fig. 2). We filmed 50 mosquitoes for 20 min in an empty room. To model the flight behavior, we consider three forces acting on a mosquito as diagrammed in fig. S2: a thrust in the direction of flight $\hat{v}$, a constant gravitational force, and a levitational force in $\hat{z}$ that counteracts gravity. In reality, a mosquito has a visual-based control system that adjusts its flight actively. Nevertheless, our results indicate that the best learned model includes a constant levitation force that precisely balances the gravitational force (fig. S2), suggesting minimal preference for the $\pm \hat{z}$ direction of flight. Therefore, we ignore these two forces in subsequent analysis. This simplifies the free-flight Langevin equation to $\frac{dv}{\mathrm{dt}}=\alpha \left(v\right)v+\xi$ (Fig. 2A), where the learned thrust α(v)v corresponds to a force derived from a speed potential U(v) with $\alpha \left(v\right)=-U^{\prime }\left(v\right)/v$.

To further examine behavioral variability, we performed learning on individual tracks and projected the thrust factor α(v) onto the two most relevant basis functions. The learned coefficients revealed two distinct clusters: one corresponding to an “active” state of flight and the other to an “idle” state (Fig. 2B). The active state tends to maintain a constant flight speed of ~0.7 m/s, with the mosquito accelerating (positive α) if its flight speed is too low and decelerating (negative α) if its speed is too high (Fig. 2C). The active state flight speed is consistent with experimental values (0.4 to 1.8 m/s) measured for other mosquito species (*12*). The idle state corresponds to a projectile thrown at an arbitrary initial speed; the mosquito does not attempt to maintain a baseline speed through accelerations or decelerations with zero α(v) (Fig. 2D), allowing the initial speed and noise to dictate the dynamics of flight . The active state exhibits higher mean speed (Fig. 2E), stronger learned noise (Fig. 2F), and longer trajectory duration (Fig. 2G) than the idle state, suggesting that the active state may reflect exploration behavior while the idle state corresponds to preparation for landing. The idle state is more often observed in trajectories near the ceiling of the chamber (fig. S5).

To validate the learned model, we simulate the learned dynamical equations with the same initial conditions as the experimental data. The simulated trajectories (Fig. 2A, bottom) qualitatively resemble the observed trajectories (Fig. 2A, top). To further quantify the similarity, we compute three time-lagged statistics, 〈S(t,t+τ)〉, which compare two time points along a trajectory separated by a lag time τ (Fig. 2, H to J): the mean squared displacement (MSD), defined by $S_{r}\left(t,t+\tau \right)={|r\left(t\right)-r\left(t+\tau \right)|}^{2}$, the directional correlation, defined by $S_{\hat{v}}\left(t,t+\tau \right)=\hat{v}\left(t\right)\cdot \hat{v}\left(t+\tau \right)$, and the speed correlation, defined by $S_{v}\left(t,t+\tau \right)=\left[v\left(t\right)-\langle v\rangle \right]\left[v\left(t+\tau \right)-\langle v\rangle \right]$. Here, 〈⋅〉 denotes the average over all time points *t* of all trajectories. The MSDs of both simulated and observed trajectories show a ballistic flight at short lag times, followed by a transition to more diffusive movement at around τ=1s (Fig. 2H). However, the experimental trajectories are not long enough to fully capture the diffusive behavior beyond this timescale. The directional correlation of mosquito flight roughly follows an exponential decay $e^{-\tau /\tau_{d}}$ (Fig. 2I), representing straight flight over short time intervals before making turns after $\tau_{d}\approx 1.3$ s. The speed correlation decreases toward zero with the lag time, a characteristic of the Langevin equation. The experimental speed correlation exhibits short-time oscillations that may be due to limitations of the tracking procedure rather than reflecting genuine insect behavior (Fig. 2J and fig. S6). Therefore, we do not aim to model this oscillatory behavior. The close agreement between the experimental data and the model simulations demonstrates that our learning framework reliably estimates the Langevin equation and behavioral forces governing mosquito flight dynamics in the absence of sensory cues.

### Mosquitoes are attracted to visual cues and CO_2_ plumes through differential responses

**Fig. 3.**
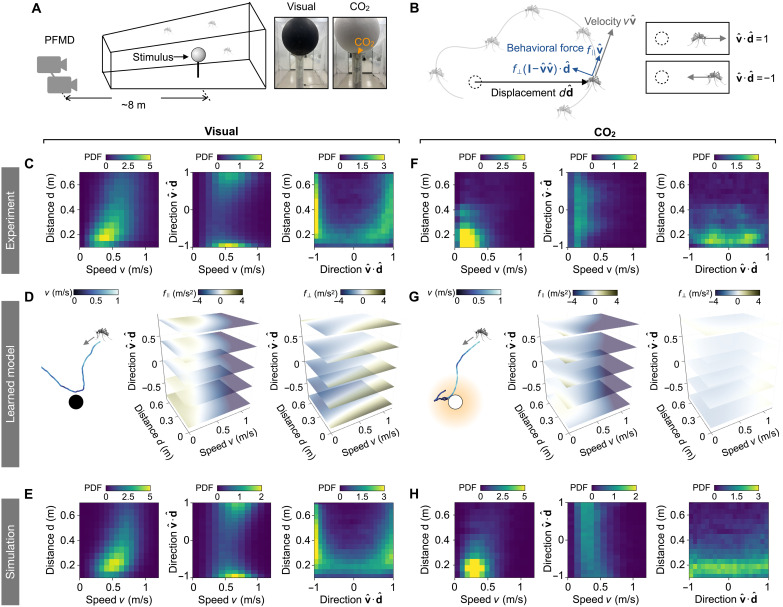
Learning dynamical models of mosquito flight behaviors reveals different responses to stimuli. (**A**) Schematics of the experimental setup. Photo subject: C.Z. Photographer: D.L.H., Georgia Institute of Technology. (**B**) Illustration of a mosquito flying with a velocity $v\hat{v}$ and a displacement $d\hat{d}$ relative to the stimulus (dashed circle). The behavioral forces are decomposed into components parallel ($f_{∥}$) and perpendicular $\left(f_{⊥}\right)$ to v and are expressed as functions of the flight speed *v*, the distance *d* to the stimulus, and the flight direction $\hat{v}\cdot \hat{d}$ (−1 toward and +1 away). (**C** to **E**) Mosquito response to a visual stimulus. (C) 2D density maps of recorded trajectories reveal mosquito attraction to the visual stimulus with bidirectional flights. (D) Left: Simulated trajectory of the learned model. Heatmaps of the learned responses (middle) $f_{∥}$ and (right) $f_{⊥}$ to visual stimulus. Yellow/blue represent speed increase/decrease ($f_{∥}$) or turning away/toward the stimulus ($f_{⊥}$). (E) 2D density maps of simulated trajectories agree quantitatively with the experimental data in (C), with a symmetric Kullback-Leibler distance $D_{\text{KL}}^{S}=0.20$ outperforming the best Gaussian fit ($D_{\text{KL}}^{S}=0.36$). (**F** to **H**) Mosquito response to CO_2_ cues. Plots correspond to (C) to (E). Simulated and experimental distributions yield a symmetric Kullback-Leibler distance $D_{\text{KL}}^{S}=0.41$, smaller than that of a Gaussian fit ($D_{\text{KL}}^{S}=0.76$).

**Fig. 4.**
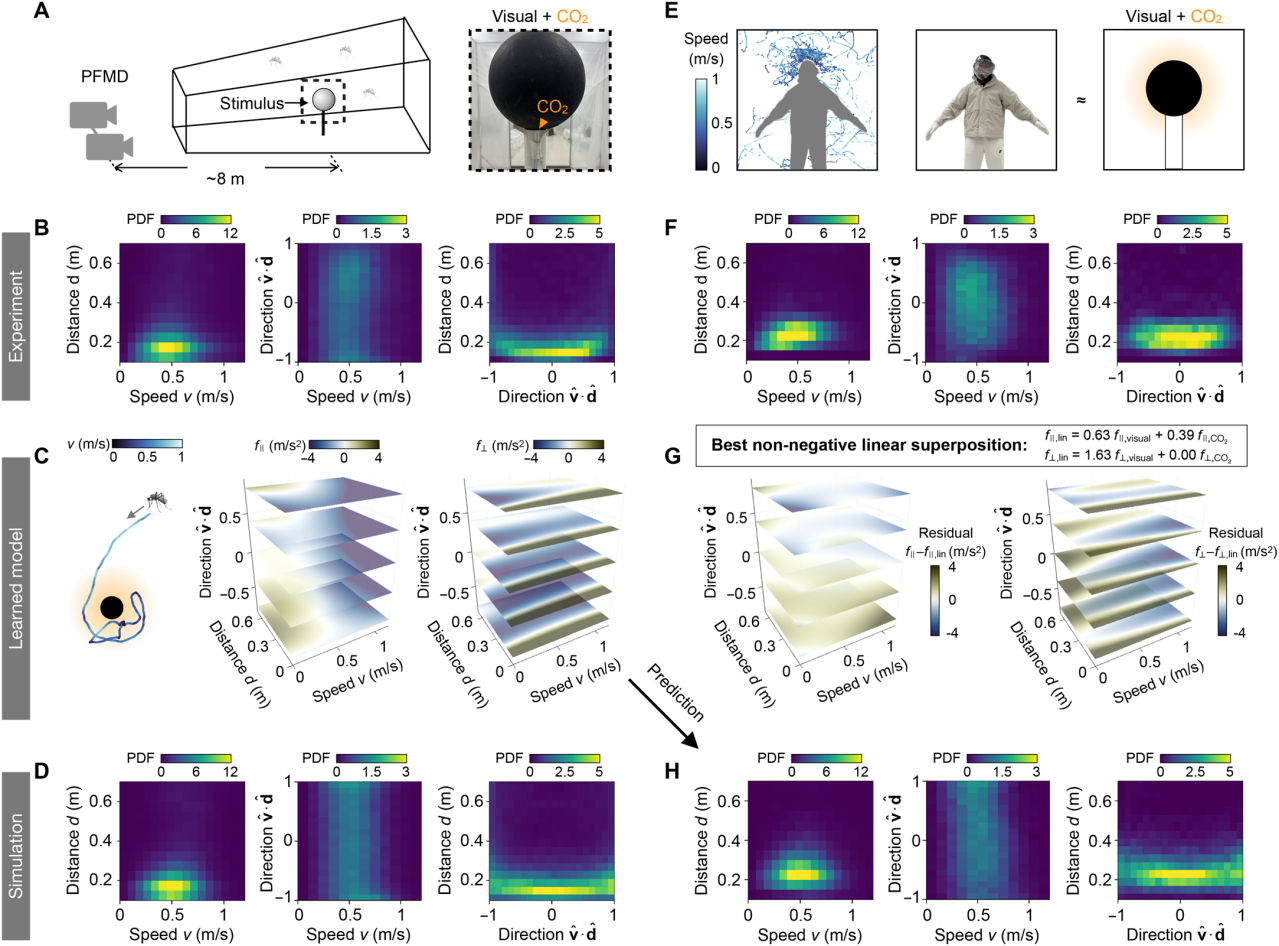
Mosquitoes combine visual and CO_2_ cues to target human hosts. (**A**) Schematics of the experimental setup. Photo subject: CZ. Photographer: D.L.H., Georgia Institute of Technology. (**B** to **D**) Mosquito response to the combined visual and CO_2_ cues. Plots correspond to Fig. 3 (C to E). A symmetric Kullback-Leibler distance of $D_{\text{KL}}^{S}=0.31$ is computed between the distributions in (B) and (D), which is smaller than that between (B) and its best Gaussian fit ($D_{\text{KL}}^{S}=0.98$). (**E**) Left: Mosquito trajectories around a human subject wearing a black hood and white outfit (shown in middle). Right: The human subject can be approximated by a black sphere emitting CO_2_. Photo subject: CZ. Photographer: D.L.H., Georgia Institute of Technology. (**F**) Heatmaps of mosquito densities at varying distances *d* from the center of the head, flight speeds *v*, and flight directions $\hat{v}\cdot \hat{d}$, for the experiment in (E). (**G**) The learned forces in (C) are approximated by nonnegative linear superposition, $f_{∥,\text{lin}}$ and $f_{⊥,\text{lin}}$, of the responses to individual cues. Heatmaps showing the difference between the learned and linearly reconstructed forces indicate that the mosquito’s response to combined stimuli is not a simple sum of uni-stimulus responses. (**H**) Heatmaps of simulated trajectories predicted by the model learned in (C). Plots correspond to (F). A symmetric Kullback-Leibler distance of $D_{\text{KL}}^{S}=0.53$ is computed between the distributions in (F) and (H), which is smaller than that between (F) and its best Gaussian fit ($D_{\text{KL}}^{S}=0.70$).

**Fig. 5.**
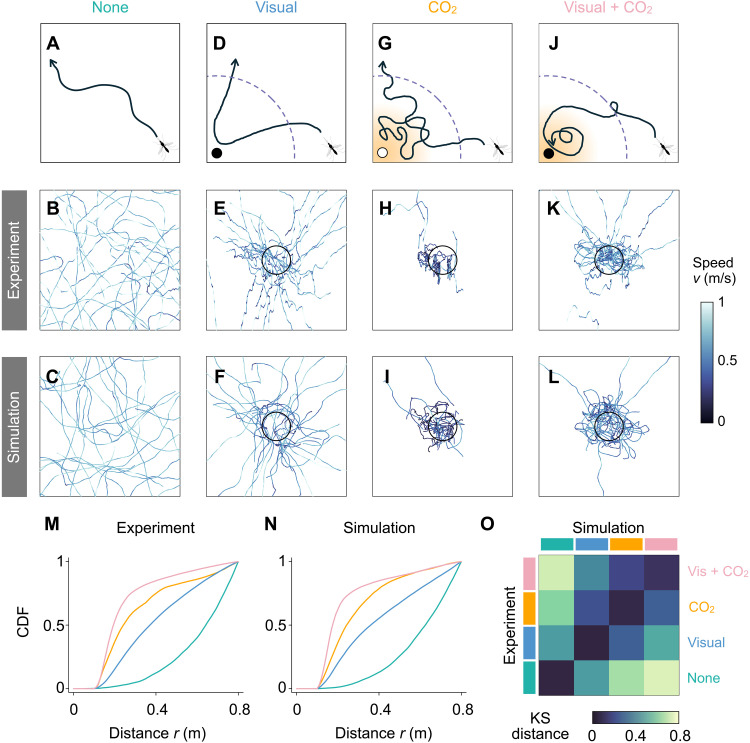
Model-predicted mosquito trajectories in response to different stimuli agree with experimental observations. (**A**) Representative trajectory of a mosquito in an empty chamber. (**B**) Typical experimental trajectories (*n* = 30) in a 1-meter cube without cues. These 3D trajectories are projected onto a 2D plane. (**C**) 2D projection of 3D simulated trajectories (*n* = 30) of mosquitoes in an empty chamber. (**D** to **L**) Schematic, experimental, and simulated trajectories of mosquitoes in response to [(D) to (F)] visual cues, [(G) to (I)] CO_2_ cues, and [(J) to (L)] combined visual and CO_2_ cues. For each cue, *n* = 30 trajectories are shown. In (D), (G), and (J), dashed circles indicate the zone of attraction, and diffuse orange denotes CO_2_. In (H) and (I), the trajectories are spread out in the third dimension. (**M** and **N**) Cumulative distribution functions (CDFs) of (M) the experimental trajectories and (N) the simulated trajectories in response to the specified sensory cues indicate that the learned models capture the key features of mosquito flight behavior. (**O**) Kolmogorov-Smirnov (KS) distances demonstrate a quantitative agreement between the experimental and simulation CDFs.

Having established a basis for mosquito behavior, we next add attractive cues into the environment. In this work, we focus on visual and CO_2_ cues (Figs. 3 to 5), but our experimental and learning framework should be directly applicable to other stimuli such as odor and heat. We use an 8-inch (≈0.2 m) diameter sphere mounted on a white pole as the attractive target for mosquitoes. To minimize boundary effects, the sphere is placed at the center of the chamber (Fig. 3A). A black sphere is used as a visual target due to its high contrast with the surrounding white walls. The CO_2_ target is a white sphere with piping releasing CO_2_ at a rate of 0.24 liters/min, comparable to the CO_2_ emission rate of human breathing (*66*).

We describe the state of a mosquito by its displacement d from the spherical target and body velocity v (Fig. 3B). The dot product $\hat{v}\cdot \hat{d}$ represents the orientation of mosquito flight, with $\hat{v}\cdot \hat{d}=-1$ indicating flight directly toward the target (taxis) and $\hat{v}\cdot \hat{d}=1$ indicating flight away from the target. To visualize mosquito flight patterns around the target, we present heatmaps of trajectory densities at varying distances *d* to the target, flight speeds *v*, and flight orientation $\hat{v}\cdot \hat{d}$ (Fig. 3, C and F).

Our data reveal distinct mosquito flight patterns in response to visual or CO_2_ cues. We consider telotaxis (movement toward) the visual cue first (Fig. 3C, left). The diffuse yellow region in the density heatmap shows slower mosquitoes concentrated in a zone of radius 0.4 m from the target. As the distance exceeds about 0.4 m, the speed distribution returns to the baseline observed in the absence of a stimulus (Fig. 2E), which is consistent with the mosquito’s visual range calculated from their eye’s minimal resolvable angle of 12.3° (*31*). The mosquito’s flight is approximately bidirectional, where mosquitoes move directly toward or away from the visual target, as shown by trajectory densities concentrated around the two zones, $\hat{v}\cdot \hat{d}=\pm 1$ (Fig. 3C, middle and right). We interpret the movement away from the target as rejection of it in the absence of essential cues—such as body odor, humidity, and heat—to induce landing and blood feeding. A closer examination of trajectories (Fig. 5E) shows that, although mosquitoes are attracted to the visual target, they often perform a “fly-by” and do not consistently engage with it due to the absence of additional host cues (movie S3). Nevertheless, the visual cue alone can attract a high density of mosquitoes. To demonstrate that attraction to black spheres can be extended to arbitrary shapes, we wrote “GT” in black using a 40-inch (1-m) font on the white floor, attracting mosquitoes approximately uniformly to each part of the lettering (fig. S7).

When presented with the CO_2_ cue, mosquitoes perform “double-takes” or tumbling behavior with decreased speed and increased turning that keeps them in the local vicinity of the target (Fig. 5H and movie S4). This tumbling has no directional preference, with $\hat{v}\cdot \hat{d}$ uniformly distributed between −1 and 1 (Fig. 3F, middle and right). CO_2_-induced tumbling is thus a form of kinesis or nondirectional response, which, in this case, increases the mosquito’s concentration near the target relative to that of a visual cue (Fig. 3F, left). Mosquitoes can sense CO_2_ concentrations as low as 103 parts per million (*67*). Numerical simulations of our experimental setup show that the zone of detectable CO_2_ extends to 0.5 m from the source (fig. S8), which aligns with our observation of increased mosquito density within 0.3 m of the target.

To model mosquito response to sensory cues, we decompose the behavioral force

$$f=f_{∥}\hat{v}+f_{⊥}\left(I-\hat{v}\hat{v}\right)\cdot \hat{d}$$ (3)

into two components depicted in Fig. 3B: a longitudinal force $f_{∥}$ along the direction of flight $\hat{v}$ and a transverse force $f_{⊥}$. The longitudinal force $f_{∥}$ represents the mosquito’s throttle, with positive and negative values corresponding to acceleration and deceleration, respectively, in the mosquito’s direction of motion. The transverse force $f_{⊥}$ represents the mosquito’s turning, with negative $f_{⊥}$ indicating turning toward the target and positive $f_{⊥}$ indicating turning away from it. We expand $f_{⊥}$ and $f_{∥}$ onto basis functions, given by tensor products of univariate scalar functions of *v*, *d*, and $\hat{v}\cdot \hat{d}$, and apply Bayesian inference to estimate the force magnitudes.

The learned forces represent different responses to visual and CO_2_ cues, which we discuss in turn. In response to visual cues, the longitudinal force $f_{∥}$ shown by the stacked heatmaps in Fig. 3D (middle) resembles a speed potential force with the preferred speed indicated by white heatmap regions where $f_{∥}$ = 0 and gradually decreasing as the mosquitoes approach the target (fig. S9). The transverse force $f_{⊥}$, shown in Fig. 3D (right), reorients mosquitoes toward the visual target when they are far from it but directs them away when they are too close, likely to prevent collisions and facilitate departure if no additional attractants are detected.

In contrast, in response to CO_2_ cues (Fig. 3G and fig. S9), $f_{⊥}$ is much weaker than its counterpart for visual cues, showing widespread white regions indicating $f_{⊥}\approx 0$ throughout. The $f_{∥}$ force corresponds to a deceleration, a slowdown from 0.7 to 0.2 m/s, when the distance to the CO_2_ source drops below 0.4 m. This deceleration alone is sufficient to explain the higher concentration of mosquito tracks near the CO_2_ stimuli (*68*). Simulations of the learned models quantitatively match the experimental data, including the density distributions around the stimuli (Fig. 3, E and H), bidirectional flight toward or away from the visual cue (Fig. 5, D to F, and movie S5), and tumbling behavior near the CO_2_ source (Fig. 5, G to I, and movie S6). This agreement is further corroborated by evaluating symmetric Kullback-Leibler distances between experimental and simulated trajectory distributions, which are smaller than those between the experimental data and their corresponding best Gaussian fits (Fig. 3, E and H, and fig. S10; see captions for numerical values of these distances). Thus, our framework provides a powerful tool for learning quantitative models of mosquito behavior in response to individual cues.

### Mosquitoes combine visual and CO_2_ cues to target human

How do mosquitoes respond to combined sensory stimuli? To explore this question, we track 3D mosquito trajectories around a black sphere releasing CO_2_ plumes (Fig. 4A), representing combined visual and CO_2_ cues. The densities of mosquito trajectories are more concentrated around the spherical target compared to experiments with either visual or CO_2_ cues alone (Fig. 4B, left), suggesting a stronger attraction to the combined stimulus. The mosquitoes maintained a flight speed of ~0.5 m/s near the target, higher than the speeds observed in the single-cue tests using CO_2_ or visual cues. Moreover, our data show a high density of trajectories with $\hat{v}\cdot \hat{d}$ close to zero, indicating that mosquitoes were more likely to circulate around the target (Fig. 4B, right). Individual trajectories further confirm the sustained orbiting behavior (Fig. 5K and movie S7). These results suggest that the combination of visual and CO_2_ cues activates a distinct behavioral state that facilitates mosquito host seeking.

To better understand this behavioral state, we apply the same Bayesian inference techniques as before to learn the $f_{∥}$ and $f_{⊥}$ forces in response to the combined cues. $f_{∥}$ shows signatures of responses to individual cues (Fig. 4C). When the mosquitoes fly toward the target (negative $\hat{v}\cdot \hat{d}$), $f_{∥}$ mirrors the response to visual cues alone, representing a speed potential force with reduced speed near the target. When mosquitoes fly away from the target (positive $\hat{v}\cdot \hat{d}$), $f_{∥}$ resembles the response to CO_2_ alone, showing a transition to extremely low speeds when the distance to the target is below 0.4 m. The $f_{⊥}$ force shows a similar pattern to that with a visual cue alone: It reorients mosquitoes toward the target when far away while directing them away from the target when in close proximity, but with a much stronger magnitude (Fig. 4C). The number of model terms that maximize the Bayesian information criteria (BIC) for both combined and individual cues is consistently around 25 terms (fig. S11). While this number depends on the choice of basis functions, it suggests comparable model complexity across these conditions. Simulations of the learned model quantitatively capture the density distributions of mosquito tracks observed in the experiment (Fig. 4D and fig. S10) and reproduce the orbiting behavior around the target (Fig. 5L and movie S8).

To assess whether the response to combined cues could be understood by adding up the individual stimulus responses, we perform nonnegative linear regression, approximating the learned force of the full response as a linear superposition $f_{\text{lin}}=\alpha_{1}f_{\text{visual}}+\alpha_{2}f_{{\text{CO}}_{2}}$ (Fig. 4G). We find that $f_{∥,\text{lin}}=0.63f_{∥,\text{visual}}+0.39f_{∥,{\text{CO}}_{2}}$, consistent with the mixed features of individual responses in $f_{∥}$, and $f_{⊥,\text{lin}}=1.63f_{⊥,\text{visual}}+0.00f_{⊥,{\text{CO}}_{2}}$, consistent with an amplified visual-driven reorientation pattern observed in $f_{⊥}$. However, the linear superposition of the force alone does not fully recapitulate the learned forces in response to combined cues, with substantial deviations between the learned and linearly reconstructed forces, particularly in regions near the target (Fig. 4G and fig. S12). Moreover, simulations using the linearly combined forces fail to reproduce the experimentally observed trajectory density around targets with combined cues. These discrepancies indicate that mosquito responses to visual and CO_2_ cues are not additive at the level of learned behavioral forces and may involve nonlinear integration and potential interactions between different sensory pathways (*6*, *11*). CO_2_ not only acts as an attractive bait for mosquitoes but also excites and activates them to be more responsive to other host-finding cues (*16*, *24*).

To further test whether the learned model generalizes beyond its training data, we predict mosquito flight behavior around a human subject wearing all white except for a black hood, our best approximation of a “spherical human” (Fig. 4E). We approximate the human subject as a 12-inch-diameter (0.3-m) black sphere emitting CO_2_, representing combined visual and CO_2_ cues. The learned behavioral force is rescaled to match the size of the human head (see the Supplementary Materials). As shown in Fig. 4 (F and H), model predictions quantitatively replicate the experimental mosquito densities around the human head, demonstrating the model’s ability to accurately describe key features of mosquito behavior in a realistic setting.

### Assessing mosquito bite risk

To prevent the spread of disease, it is important to keep track of the potential bite risk, which we measure as the distance $d_{50}$ at which 50% of mosquito trajectories are concentrated near an attractive target. Thus, a smaller $d_{50}$ value indicates a closer proximity of flying mosquitoes. For an 8-inch (≈0.2-m) spherical target, Fig. 5 (M and N) shows the cumulative distributions of mosquitoes as a function of distance from various targets, for both experimental data and simulations of the learned models. The $d_{50}$ values are roughly 0.65 m for no cues, 0.4 m for visual cues, 0.25 m for CO_2_ cues, and 0.2 m for combined visual and CO_2_ cues. The close agreement between the experimental and simulated data, as shown by the Kolmogorov-Smirnov distance in Fig. 5O, again highlights the accuracy of our model in predicting mosquito behavior under various conditions.

## DISCUSSION

In this study, we developed protocols and a model learning pipeline for quantitatively characterizing mosquito responses to host-linked cues, such as visual and CO_2_ cues. To our knowledge, we have generated one of the largest mosquito 3D tracking datasets to date (tables S1 and S2), which enabled the identification of dynamical models for mosquito behavior in the presence of different environmental cues. We find that a combination of visual and CO_2_ cues attracts more mosquitoes than individual cues alone. Our results provide valuable insights into mosquito flight patterns around human subjects. For instance, our experiments confirm that *Ae. aegypti* mosquitoes show a preference for darker colors and primarily target the head of a human (Figs. 1C and 4E), which present both visual and CO_2_ cues. Beyond capturing these flight patterns, our model also provides a compact representation of mosquito behavior by summarizing thousands of trajectories in less than 30 parameters while filtering out both biological and measurement noise. To make these insights accessible to a broader audience, we have developed an interactive web application that incorporates all the learned mosquito models (*69*). This application allows users to change the type of attractive cues and the number of mosquitoes and to visualize mosquito flight dynamics (fig. S13).

Most previous experiments on mosquito host-finding have been conducted in wind tunnels where emitted CO_2_ triggers mosquitoes to fly upwind toward the source (*6*, *15*, *22*, *24*). Our study of mosquitoes flying in a closed room is most similar to studies of mosquitoes flying downwind to hosts (*13*), a situation that has received less attention. In such conditions, odor and CO_2_ cues stay relatively stationary, consistent with our observation that vision is an important part of host-finding for *Aedes* mosquitoes.

While we primarily focused on understanding mosquito responses to a single target, we have also conducted preliminary experiments to explore how mosquitoes respond to multiple targets (figs. S14 and S15). Developing a quantitative framework for how mosquitoes integrate cues from multiple targets and make decisions will be an important future step toward predicting mosquito behavior around typical humans, beyond the simplified spherical human in our study (Fig. 4E). Our study of combined sensory cues provides a first step in this direction, laying the groundwork for understanding mosquito behavior in complex environments such as those involving human groups. Additionally, our current experimental setup focused on flight behavior. The PFMD used here is not effective at collecting trajectories where mosquitoes land and blood feed. Incorporating these aspects in future experimentation will be crucial for developing a comprehensive understanding of mosquito interactions with hosts.

Through combined experiments and data-driven modeling, we identified the temporal dynamics of mosquito flight in response to visual and CO_2_ cues. One potential application of our framework is optimizing the design of mosquito suction traps by exploiting these temporal dynamics. Now, most suction traps rely on steady cues, such as continuous CO_2_ release or constant light sources, to attract mosquitoes (*22*, *70*). Incorporating temporal modulation of cues, such as presenting intermittent visual cues and CO_2_ pulses and activating suction at intervals, may help reduce the target rejection behavior observed when mosquitoes approach the trap and detect the absence of human-associated cues. Our framework can be used to learn generative models of mosquito flight dynamics in the presence of airflow near suction traps. This will allow us to test and optimize temporally modulated trap designs in silico before experimental implementation.

Last, we expect our framework to be widely applicable for quantifying the behavior of other mosquito species, such as the malaria mosquito *Anopheles gambiae*, as well as the behavioral changes caused by pathogen infections in mosquitoes and other vectors (*71*, *72*). These future studies could guide the development of effective strategies for surveillance, capture, and repellence of mosquitoes and reduction in mosquito-borne diseases.

## MATERIALS AND METHODS

The methods are summarized here and expounded upon further in the Supplementary Materials.

### Experimental materials and methods

In this study, we conducted experiments on female *Ae. aegypti* mosquitoes that were 3 to 5 days old (postemergence). We used a 3D infrared camera system, the PFMD to capture high-resolution insect trajectories. The system uses a dual-lens camera, infrared LEDs, and a retroreflective backdrop to capture precise trajectories at a time step of 0.01 s (*50*). After obtaining the trajectory data from the PFMD system, we filtered out trajectories within the initial 5 min of the experiment, trajectories shorter than 1.0 s, and trajectories close to the walls. Eliminating these trajectories mitigated effects from the release of mosquitoes, reduced noise in the trajectory data, and reduced effects of mosquitoes landing on the walls of the chamber.

We conducted three types of experiments, including free flight, human mimic, and human experiments. These experiments are conducted under Georgia Institute of Technology’s Institutional Review Board protocol H23325 ensuring adherence to ethical standards and guidelines. To obtain informed consent, we provided volunteers with a document listing the procedures, risks, and benefits for participating in our experiments. Consent was provided by signature. In the free flight experiments, we released 100 mosquitoes into an empty chamber for 20 min. The data collected from this experiment were used to validate our framework for learning dynamical models for mosquito flight. For the human mimic experiments, we used 4-, 8-, 12-, and 16-inch-diameter (0.10-, 0.20-, 0.30-, and 0.41-m, respectively) expanded polystyrene spheres painted either black or kept bare (white) as mimics for human targets. In all the experiments, the sphere was elevated 5 ft (152 cm) off the ground and placed 300 cm away from the release point of the mosquitoes. To compare mosquito preferences between two targets, a T-joint was added to the 5 ft (152 cm) stand, and experiments were conducted with two 4-inch (0.10-m) spheres, a 4- and 8-inch (0.10- and 0.20-m, respectively) sphere, a 4- and 12-inch (0.10- and 0.30-m, respectively) sphere, and a 4- and 16-inch (0.10- and 0.41-m, respectively) sphere. To study the effects carbon dioxide, we released CO_2_ from a tank with a calibrated flow meter controlled to have a volumetric flow rate of 0.24 liters/min matching the rate of human breathing (*66*). The CO_2_ experiments were conducted with the 8-inch (0.20-m) sphere. The human experiment was conducted with a human target wearing a series of outfits, including “casual clothes” (a dark sweatshirt and jeans outfit), a half-black, half-white outfit, and a white outfit with a black wrapping shown in Figs. 1B, 1C, and 4E, respectively.

### Bayesian inference of mosquito flight dynamics

Introducing Eq. 2 into Eq. 1 and stacking all the trajectories from a single experiment into matrix form, we obtained $\dot{v}=\mathrm{\Theta w}+\xi$. Here, each row corresponds to a spatial component of a mosquito trajectory at a specific time point, each column of Θ corresponds to a basis element $\theta_{m}$ in Eq. 2, and the coefficients $w_{m}$ are collected in a vector w. To perform Bayesian inference of w, we minimized the negative log-posterior

−lnP(w∣data)=−lnP(data∣w)−lnP(w)+const (4)

with respect to w given the experimental data, where P(data∣w) is the likelihood and −lnP(w) is the prior probability. The negative log-likelihood function is given by $-\text{ln}P\left(\text{data}|w\right)=\frac{N}{2}\text{ln}\left(2\pi \right)+\frac{1}{2}\text{ln}|\Psi |+\frac{1}{2}{\left(\dot{v}-\mathrm{\Theta w}\right)}^{T}\Psi^{-1}\left(\dot{v}-\mathrm{\Theta w}\right)$, where $\Psi =\Delta I_{N}$ is a diagonal matrix assuming Gaussian white noise (Eq. 1) and *N* is the number of rows Θ contains. As described in the section “Learning dynamical models for mosquito flight,” we used a sparsity-promoting Gaussian prior, leading to a negative log-prior $-\text{ln}P\left(w\right)=\sum_{m}\frac{w_{m}^{2}}{2\gamma_{m}}+\sum_{m}\frac{1}{2}\text{ln}\left(2{\pi \gamma }_{m}\right).$ Combining these expressions, we obtained a posterior that follows a Gaussian distribution P(w∣data)=N(w;μ,Σ), where the covariance matrix **Σ** and the mean **μ** are given by $\Sigma ={\left(\Theta^{T}\Psi^{-1}\Theta^{T}+\Gamma^{-1}\right)}^{-1},\mu ={\Sigma \Theta }^{T}\Psi^{-1}\dot{v},$ and Γ is a diagonal matrix with diagonal element $\Gamma_{\mathrm{mm}}=\gamma_{m}$. To determine the values of Δ and $\gamma_{m}$, we followed previous work (*40*) and used the expectation maximization approach to iteratively update our estimates of $\gamma_{m}$ and Δ. Specifically, using $\gamma_{m}^{\left(n\right)}$ and $\Delta^{\left(n\right)}$ from the previous iteration, we updated their current estimates as follows: $\gamma_{m}^{\left(n+1\right)}=E_{w∼N\left(\mu^{\left(n\right)},\Sigma^{\left(n\right)}\right)}\left[w_{m}^{2}\right]={\left(\mu_{m}^{\left(n\right)}\right)}^{2}+(\Sigma_{\mathrm{mm}}^{\left(n\right)})$ and $\Delta^{\left(n+1\right)}=E_{w∼N\left(\mu^{\left(n\right)},\Sigma^{\left(n\right)}\right)}\left[\frac{{|\dot{v}-\mathrm{\Theta w}|}^{2}}{N}\right]=\frac{1}{N}\left[{|\dot{v}-{\Theta \mu }^{\left(n\right)}|}^{2}+\Delta^{\left(n\right)}\sum_{m}\left(1-\Sigma_{\mathrm{mm}}^{\left(n\right)}/\gamma_{m}^{\left(n\right)}\right)\right]$.

## Supplementary Materials

### The PDF file includes:

Supplementary Text Figs. S1 to S18 Tables S1 and S2 Legends for movies S1 to S8 References
