# Supplementary material for: Predicting mosquito flight behavior using Bayesian dynamical systems learning
Source: Sci Adv. 2026 Mar 18;12(12):eadz7063. doi: 10.1126/sciadv.adz7063 (PMC12998517; doi:10.1126/sciadv.adz7063)
Supplement: Supplementary file 1 — Supplementary Text Figs. S1 to S18 Tables S1 and S2 Legends for movies S1 to S8 References [file sciadv.adz7063_sm.pdf]

Science Advances
AAAS

Supplementary Materials for

Predicting mosquito flight behavior using Bayesian dynamical systems learning

Christopher Zuo *et al.*

Corresponding author: Jörn Dunkel, dunkel@mit.edu; David L. Hu, hu@me.gatech.edu

Sci. Adv. **12**, eadz7063 (2026)
DOI: 10.1126/sciadv.adz7063

The PDF file includes:

Supplementary Text
Figs. S1 to S18
Tables S1 and S2
Legends for movies S1 to S8
References

Other Supplementary Material for this manuscript includes the following:

Movies S1 to S8

Supplementary Text

1 Experimental materials and methods

1.1 Mosquito rearing

In this study, we focus on the anthropophilic mosquito *Aedes aegypti*, which feeds during the day and is a vector of several pathogens. The mosquito strain used in this study was obtained through BEI Resources, NIAID, NIH: *Aedes aegypti*, Strain ROCK, MRA-734, contributed by David W. Severson. All mosquito rearing took place in an insect rearing room (Bahnson Environmental Chamber CCS-300, Clemmons, NC) at 27°C and 78% humidity with 12:12 light and dark cycles that include 30-minute periods of sunrise/sunset.

For each trial, a cohort of about five hundred mosquitoes were reared and then the female mosquitoes (7-21 days post emergence) were fed defibrinated rabbit blood (Hemostat Labs, DRB050, Dixon, CA) using a membrane feeder. Three days post-blood feeding, oviposition cups were placed inside each cage. The oviposition cup was a 150-ml cup lined with seed germination paper (Anchor Paper Company, SD7615L, St. Paul, MN) with purified water covering the bottom of the cup (depth 0.50 cm). *Aedes* species lay eggs on the inner wall of containers and just above the water line. By placing the seed germination paper around the inner wall of the cup, the mosquitoes lay eggs on the paper creating an egg sheet. Twenty-four hours later after placing it into the cage, the oviposition cup was removed. Excess water, eggs not adhered to the egg sheets, and dead adults were gently rinsed from the oviposition cup, and the cup with egg sheets was placed in a closed Tupperware container. It remained in the closed container for 72 hours and then the lid was cracked 1 cm to allow for water evaporation. Once the egg sheets were completely dry (48-72 hours later), egg sheets were removed from the cup and stored in plastic Ziploc bags. *Aedes* eggs can be stored in this manner for several months and still remain viable.

Two weeks prior to the scheduled experiment date, an egg sheet was removed from the Ziploc storage bag, placed in a petri dish and submerged in water and then placed in a vacuum chamber for 15 minutes at 15 psi. The egg sheet was then placed in a larval rearing pan (US Plastics, 52051, Lima, OH) with 250 ml purified water and 25 mg ground fish diet (Tetra, TetraMin Tropical Granules)

to allow eggs to hatch and larvae to emerge. Larval rearing pans were placed in an insect rearing room (Bahnson Environmental Chamber CCS-300, Clemmons, NC) at the previously mentioned rearing conditions. Larvae were kept at a density of approximately 200 larvae per rearing pan and fed 4-6 pellets of high-quality koi food daily following standard *Aedes* rearing protocols. The larval pan water was cleaned daily. Pupae were put in a 150 ml plastic cup and placed in a Bug Dorm (MegaView Science, DP-1000, Taichung, Taiwan) with a hanging sugar vial (10% sugar, 0.1% methylparaben). Once all pupae had emerged into adults, the pupae cup was removed. The sugar vial was monitored daily to ensure it remained at a sufficient volume. Mosquitoes are stored in environmental chambers until the day of the experiment. On the day of the experiment, mosquitoes are sorted manually and transferred from the storage cages into release capsules using mouth aspirators.

1.2 Capturing Trajectories

To attain mosquito flight trajectories, we worked with the Centers for Disease Control and Prevention (CDC) in Atlanta to employ a state-of-the-art 3-D infrared camera system, the Photonic Fence Monitoring Device (PFMD). The PFMD system, developed by Photonic Sentry, is a specialized imaging device designed to detect and track flying insects with unprecedented precision and speed. The system uses a dual-lens camera, infrared LED's, and a retro-reflective backdrop for precise position tracking of mosquitoes at time steps of 0.01 seconds (*50*).

After obtaining trajectories from the PFMD, we filter to remove early time trajectories to mitigate effects from the mosquito release, short time trajectories, and trajectories near the walls of the chamber to eliminate effects from mosquitoes landing on walls. To eliminate effects from mosquito release, we remove all trajectories within the first 5 minutes of the experiment. Furthermore, we remove all trajectories that are shorter than 1.0 second, as suggested by the PFMD manufacturers, to reduce noisy trajectories. Finally, to remove trajectories from mosquitoes landing on the walls, we identify the walls by finding the 1st and 99th percentile of all mosquito positions in each dimension, and we remove mosquitoes within 10 cm of the side walls, within 10 cm of the top wall and floor, and with 50 cm of the front and back walls.

The photonic sentry system employs two cameras that use stereo vision to determine the 3D

position of mosquitoes. The PFMD's accompanying software cleans the raw data and outputs trajectory data which we provide in a data depository (*73*). Although PFMD does not publish details of their data-cleaning algorithm, some filtering is applied to the raw measurements (*52*). We note that some experimental trajectories show "jitters" in the y direction (away from the camera), for example in Fig. 5E, H, and K. We hypothesize that this may be due to small errors in stereo disparity. However, our learning procedure is designed to be robust to such noise, allowing us to learn isotropic dynamical models that capture mosquito behavior without overfitting to the jitters in the experimental trajectories.

1.3 Experimental Setup

For the experiment, a chamber was set up with mesh netting extending 502 cm from a 162 cm by 152 cm clear acrylic panel to a 312 cm by 314 cm retro-reflective sheet, creating a large trapezoidal prism shown in main Fig. 1. The chamber floor was measured at 438 lux under fluorescent ceiling lights. The PFMD camera is set up across the room adjusting the focal length and the infrared LED brightness to fully focus and illuminate the contained trapezoidal prism. A plastic tarp covers the chamber to minimize external flows in the chamber. The environmental chamber is set at 28 degrees Celsius and 45 percent humidity. Tests are conducted with 50 or 100 mosquitoes and last 10 to 20 minutes.

Validation of PFMD: We validate the PFMD by performing swinging pendulum experiments with a 150 cm fishing line tied to a metal M12 hex nut. The ensuing trajectories shown by the PFMD are well-matched by predictions made of a simple nonlinear pendulum model (Fig. S16). We modeled the pendulum motion in the y-z plane using the measured pendulum length, gravity, and neglecting aerodynamic effects. The only fitting parameters are the pivot point and the initial angle and velocity. Both of these parameters were approximately measured and allowed to have small deviations from the measurement during the fitting. Despite our use of a very simple pendulum model, the predicted and measured motion show close agreement (Fig. S16). The average positional error between the experiment and model is calculated to be $\approx$ 10 cm. While this is larger than the advertised trajectory uncertainty of ±2 mm, the model error in this experiment will be much larger than the measurement error, because the setup has many difference from an ideal pendulum (air

resistance, non-fixed pivot point, not-stiff pendulum). However, this experiment gives confidence that our installed PFMD device appears to be well-calibrated and working properly because the pendulum tracks closely follow the modeled trajectory (Fig. S16). In addition, the PFMD was installed at the CDC in 2020 and also underwent its own calibration tests performed by technicians.

Commercial infrared cameras use infrared LEDs with a peak wavelength of around 860 nm. While previous work has shown that mosquitoes respond to thermal infrared (due to human body heat) (*75*) as well as wavelengths up to 700 nm (*11, 31*), studies on their vision have generally shown they are not sensitive to wavelengths in the infrared spectrum that cameras typically emit (*76, 31*). Thus, while it is possible that infrared light could affect mosquito flight, we did not observe any preferential behavior in our mosquito experiments towards the camera light source.

Depth uncertainty analysis: Depth in a stereo imaging system like PFMD is estimated from the disparity between corresponding features in the two camera views. For a standard parallel stereo configuration, the depth y (main Fig. 1A) of an object is given by $y = fB/d$, where f is the focal length, the baseline B is the distance between the optical centers of the two cameras, and the disparity d is the distance of the same object between the left and right images. To estimate the depth uncertainty Δy, we propagate a small disparity uncertainty Δd and obtain $\Delta y = |\Delta d| y/d = |\Delta d| y^2/(fB)$. We note that since $\Delta y \sim y^2$, the depth resolution is generally better for trajectories in the front of the chamber than in the back. In our PFMD configuration, the typical working distance during experiments is $y = 8$ m, and the camera baseline is $B \approx 250$ mm (roughly half the PFMD's 491 mm width). The spatial sampling interval R in the object plane is estimated from the field of view, with a 2048×2048 pixels image spanning approximately 3 m $\times$ 3 m, leading to $R \approx 1.46$ mm/pixel. Using the relationship $y/f = R/r$, where r is the pixel size of the camera sensor, the depth uncertainty can be rewritten as $\Delta y = |\Delta d| y/d = (|\Delta d|/r)(y/B)R$. This means a disparity uncertainty of 1 pixel (i.e., $|\Delta d|/r = 1$) would lead to a depth uncertainty of $\Delta y = 1 \times (8000 \text{ mm}/250 \text{ mm}) \times 1.46 \text{ mm} \approx 47$ mm.

We further performed a direct disparity measurement using synchronized left/right image pairs at the working distance $y = 8$ m, which yielded $d \approx 170$ pixels (Fig. S17). Thus, using $\Delta y = |\Delta d| y/d$, we obtain that a disparity uncertainty of $\Delta d = 1$ pixel corresponds to a depth uncertainty of $\Delta y = |\Delta d| y/d = 8000 \text{ mm}/170 \approx 47$ mm, which is consistent with the first estimate.

In addition to the optical depth error derived above, we also estimated the *effective* depth

uncertainty after trajectory denoising as follows: we simulated mosquito flight trajectories with known ground truth $y_0(t)$, added Gaussian white noise with $\sigma \approx 50$ mm to mimic the PFMD's optical depth error, and then applied spectral denoising to obtain denoised trajectories $\hat{y}(t)$. As shown in Fig. S18A, the denoised trajectory closely recovers the true trajectory, and the root mean squared error between the denoised and true depth decreases with increasing number of time points N of a trajectory as $N^{-1/2}$, consistent with averaging independent noise. Since we only analyzed trajectories longer than 1 s (i.e., $N > 100$), we estimated the effective depth error to be 10 mm or less (Fig. S18B), which is in line with PFMD's advertised depth accuracy of ±5 mm. While the PFMD's internal denoising pipeline is not fully publicly documented, and we do not know the actual size of the image PFMD captures for tracking, this numerical experiment demonstrates that denoising of the raw disparity-limited signal is sufficient to reduce the actual depth uncertainty well below the optical bound (assuming relatively smooth trajectories, which are learned with our model). Indeed, the depth trajectories output by the PFMD (Fig. S1 and S18C) are considerably smoother than the synthetic trajectories with $\sigma \approx 50$ mm noise, suggesting that an internal denoising or smoothing step is applied before data export (*77*).

Finally, we note that a similar analysis can be used to estimate the in-plane tracking error. In particular, once the depth y is determined, the in-plane coordinate x (and similarly for z) is given by $x = (u - c_u)y/f$, where u is the horizontal coordinate of the target's image on the camera sensor, c_u is the coordinate of the principal point of the camera sensor, and f is the focal length. Error propagation leads to $\Delta x = \left[(\Delta u(y/f))^2 + ((u - c_u)\Delta y/f)^2\right]^{1/2}$. From the analysis above, we know that for a working distance of $y = 8$ m, the disparity is $d \approx 170$ pixels. Thus, using $y = fB/d$ and $B \approx 250$ mm, we obtain $f \approx 5440$ pixels. For an uncertainty of $\Delta u = 1$ pixel, $\Delta u(y/f) = (8000 \text{ mm}/5440) \approx 1.47$ mm. Assuming the image is 2048×2048 pixels, we estimate the maximum of $(u - c_u)$ to be 1024 pixels, and so the maximum $(u - c_u)\Delta y/f \approx (1024/5440) \times 47 \text{ mm} = 8.85$ mm, much larger than $\Delta u(y/f)$. Thus, the maximum in-plane uncertainty Δx_{max} can be approximated by $\Delta x_{\text{max}} = (1024/5440)\Delta y \approx 0.2\Delta y$. Again, since the effective depth uncertainty after denoising can be 10 mm or less for the trajectories we analyzed, the effective in-plane uncertainty should be roughly 20% of that, i.e., 2 mm or less, consistent with the reported accuracy in PFMD's specification sheet (*77*).

Free flight: To understand mosquito behavior in the environmental chamber, mosquitoes are trans-

ferred from their holding container (container with food) into a release canister using a mosquito aspirator. The mosquitoes are sorted visually based on anatomical features. This is typically done by assessing both body size and antennal morphology: male mosquitoes are generally smaller in size, with feather-like antennae, whereas females are larger in size, with thinner, less hairy antennae. This method has been commonly used in previous studies for sex identification (*78*). Then the female mosquitoes are placed into a release canister. They are then released from a cylindrical canister (3 inches tall, 1 inch diameter) by knocking it over and having the lid fall off. The initial shock ensures that a large majority of the mosquitoes fly out of the capsule. The release into an empty chamber and their subsequent behavior is recorded with the PFMD camera. To characterize the mosquito behavior in the empty chamber, experiments were conducted with 100 mosquitoes for 20 min. These datasets are used to validate our framework for learning dynamical models for mosquito flight behaviors (see main Fig. 2 and Fig. S2).

Human mimic experiments: To introduce more cues to the chamber (visual and CO_2), we performed experiments with styrofoam spheres as visual cues, with and without emitting carbon dioxide.

We used 4, 8, 12, and 16 inch-diameter styrofoam spheres to determine how behavior depends on target size. The balls were either painted black or kept bare (white). For all experiments, the ball was elevated 5 ft off the ground, which is the midpoint between the ceiling and floor. The target is placed 300 cm away from the mosquito release point (800 cm away from the camera).

To further understand how the visual cue affects the mosquito behavior, a preference test is conducted (Fig. S15). Sphere preference testing is conducted by creating a T joint on top of the 5-ft stand, allowing for the placement of two spheres on either side of the stand. Tests were conducted with two 4-inch spheres, a 4 and 8-inch sphere, a 4 and 12-inch sphere, and a 4 and 16-inch sphere.

To study the effect of carbon dioxide, tests were conducted with spheres atop a carbon dioxide release. The carbon dioxide is released from a CO_2 tank and controlled with a calibrated flow meter. A volumetric flow rate of 0.24 L/min is released from the 0.25-inch diameter tube. The flow rate matches the rate of a human breathing. The CO_2 experiment is conducted with the 8-inch sphere.

Human experiment:

Humans are an optimal bait source as many mosquito species have evolved specifically to target humans (*3*). Using the PFMD camera and a human target, a clear silhouette is created with the

mosquito trajectories around the target in the dark clothing experiment. The target is posed as shown in Fig. 1B and C where each limb can be clearly seen in a silhouette.

We perform 3 experiments with the same human subject, but each wearing different outfits. In the first experiment, the target is wearing a dark gray fleece sweatshirt, blue jeans, cotton socks, and dark colored lab gloves, emulating a human target wearing dark colored clothing. This outfit is shown in Fig. 1B. In the second experiment, the human target wears clothes such that one side is white and the other is black, allowing for visual asymmetry shown in Fig. 1C. The top is a fleece sweatshirt where a cotton t-shirt is sewn into the hood to match the white side of the sweatshirt. The bottom is a pair of jeans where half of it is white and the other half is black. Finally, in the white torso and black head experiment, the hood is from a black fleece sweatshirt, where the torso of the sweatshirt is covered by a white polyester jacket. For the bottom the human target is wearing white fleece sweatpants and white shoes. This outfit is shown in Fig. 4E. In each of these experiments, precautions were taken to protect the target's face from mosquito bites by sewing/taping a white polyester mosquito mesh to the front opening of the sweatshirt hood. To run the experiments, the human target gets into position at the 800 cm mark from the camera and uses a long white PVC to knock down the release capsule, in doing so releasing the mosquitoes. The human target will stand still in their pose to allow for clear tracking of mosquitoes and the formation of a silhouette.

2 Mosquito swarming analysis

While we did not find any evidence of mosquito swarming behavior in the data, we did find a small number of trajectories in each dataset in which pairs of mosquitoes follow very similar paths (Fig. S1). These trajectories only occurred for mosquito pairs and numbered in the 1-10 range out of 10,000s. Currently, we do not know the cause of these trajectories but we believe they are due to mosquitoes avoiding each other or mosquitoes having similar behavioral reactions to cues.

3 Bayesian inference of dynamical models

To describe mosquito flight behavior, we use the following equations to model the temporal changes in mosquito positions $\mathbf{r}$ and flight velocities $\mathbf{v}$:

$$\dot{\mathbf{r}} = \mathbf{v}, \quad \dot{\mathbf{v}} = \mathbf{f}(\mathbf{v}, \mathbf{r}) + \boldsymbol{\xi}, \tag{S1}$$

where the dot symbol ($\dot{}$) denotes time derivative, and $\mathbf{f}(\mathbf{v}, \mathbf{r})$ is the total forces that mosquitoes experience during their flight. The Gaussian white noise $\boldsymbol{\xi}$ satisfies $\boldsymbol{\xi}(t)\boldsymbol{\xi}(t') = \Delta\mathbf{I}\delta(t - t')$, where Δ is the magnitude of the noise, $\mathbf{I}$ is an identity matrix of size 3, and δ denotes the Kronecker delta function. Our goal is to learn the behavioral forces of mosquitoes directly from 3D time-series measurements of mosquito trajectories $\mathbf{r}(t)$.

3.1 Representation of mosquito behavioral forces

To model the total forces of free-flying mosquitoes in the absence of sensory cues, we decompose $\mathbf{f} = \alpha(v)\mathbf{v} + f_z(v, \hat{\mathbf{v}}\cdot\hat{\mathbf{z}})\hat{\mathbf{z}} - g\hat{\mathbf{z}}$ into three different components: a thrust force $\alpha(v)\mathbf{v}$ in the direction of velocity $\mathbf{v}$, a levitation force $f_z(v, \hat{\mathbf{v}}\cdot\hat{\mathbf{z}})\hat{\mathbf{z}}$, and a gravitational force $-g\hat{\mathbf{z}}$ with a constant $g \approx 10\,\mathrm{m/s^2}$. Here, we use the hat symbol ($\hat{}$) to denote unit vectors, and we use non-bold letters to denote the magnitude of a vector. We note that the levitation force is assumed to be independent of the absolute height z. The dot product $\hat{\mathbf{v}} \cdot \hat{\mathbf{z}}$ describes the direction of mosquito flight, with positive values indicating upward flight and negative values indicating downward flight. We approximate $\alpha(v)$ by a linear combination of basis functions

$$\alpha(v) = \sum_m w_m \theta_m(v; v_0), \tag{S2}$$

where $\theta_m = L_m(v/v_0)\exp(\frac{-v}{2v_0})$ represents Laguerre polynomials L_m with exponential weighting factors, and the weights w_m encode the information about $\alpha(v)$ that we aim to learn from data. Here, v_0 is a speed scale parameter and its determination is described in Section 3.2 **Sparse Bayesian inference**. Similarly, we expand the magnitude of the levitation force $f_z(v, \hat{\mathbf{v}}\cdot\hat{\mathbf{z}})\hat{\mathbf{z}}$ as

$$f_z(v, \hat{\mathbf{v}}\cdot\hat{\mathbf{z}}) = \sum_\mu w_\mu \vartheta_\mu(v, \hat{\mathbf{v}}\cdot\hat{\mathbf{z}}) = \sum_{\mu_0}\sum_{\mu_1} w_{(\mu_0,\mu_1)}\theta_{\mu_0}(v; v_0)\theta^*_{\mu_1}(\hat{\mathbf{v}}\cdot\hat{\mathbf{z}}), \tag{S3}$$

where each basis function ϑ_μ is decomposed into the product of univariate functions θ and θ^*. Here, θ represents the same weighted Laguerre polynomials as described above, and θ^* denotes the

Legendre polynomials. As shown in Fig. S2, the learned levitation force has a constant magnitude $f_z = g$ that balances the gravitational force. Therefore, we ignore these two components in our analysis below.

To model the behavioral forces that mosquitoes use to maneuver their flight in response to environmental stimuli, we consider point-source stimuli and decompose the force $\mathbf{f} = f_{\|}\hat{\mathbf{v}} + f_{\perp}(\mathbf{I} - \hat{\mathbf{v}}\hat{\mathbf{v}}) \cdot \hat{\mathbf{d}}$ into two orthogonal components (see main Fig. 3): one parallel to the direction of flight $\hat{\mathbf{v}}$, and one perpendicular to it. Similar to the procedures above, we represent the force magnitudes $f_{\|}$ and $f_{\perp}$ using basis-function expansions:

$$f_{\|}(v, d, \hat{\mathbf{v}} \cdot \hat{\mathbf{d}}) = \sum_{\mu_0,\mu_1,\mu_2} w^{\|}_{(\mu_0,\mu_1,\mu_2)} \theta_{\mu_0}(v; v_0)\theta_{\mu_1}(d; d_0)\theta^*_{\mu_2}(\hat{\mathbf{v}} \cdot \hat{\mathbf{d}}), \tag{S4a}$$

$$f_{\perp}(v, d, \hat{\mathbf{v}} \cdot \hat{\mathbf{d}}) = \sum_{\mu_0,\mu_1,\mu_2} w^{\perp}_{(\mu_0,\mu_1,\mu_2)} \theta_{\mu_0}(v; v_0)\theta_{\mu_1}(d; d_0)\theta^*_{\mu_2}(\hat{\mathbf{v}} \cdot \hat{\mathbf{d}}), \tag{S4b}$$

where $\mathbf{d}$ denotes the displacement vector from the point stimulus to the mosquitoes. Again, v_0 and d_0 are speed and length scale parameters, respectively, and their determination is described in Section 3.2. In the notation of main Eq. (2), $\mathbf{f} = \sum_\mu w_\mu \boldsymbol{\theta}_\mu$, the compound index is $\mu = (\mu_0, \mu_1, \mu_2, s)$ with $s = \{\|, \perp\}$, so that $w_\mu = w^s_{(\mu_0,\mu_1,\mu_2)}$, and the vector basis $\boldsymbol{\theta}_\mu$ is given by $\boldsymbol{\theta}_\mu = \theta_{\mu_0}\theta_{\mu_1}\theta^*_{\mu_2}\mathbf{e}_s$ where $\mathbf{e}_{\|} = \hat{\mathbf{v}}$ and $\mathbf{e}_{\perp} = (\mathbf{I} - \hat{\mathbf{v}}\hat{\mathbf{v}}) \cdot \hat{\mathbf{d}}$.

3.2 Sparse Bayesian inference

To learn the behavioral forces directly from experiments, we first compute $\mathbf{v}$ and $\dot{\mathbf{v}}$ from the measurements of $\mathbf{r}(t)$ by using finite-difference methods. This allows us to construct the basis functions described in Eqs. (S2)-(S4). Introducing these expressions into Eq. (S1) and stacking all the trajectories into a matrix form, we obtain $\dot{\mathbf{v}} = \boldsymbol{\Theta}\mathbf{w} + \boldsymbol{\xi}$, which reduces the learning task into a linear regression problem. Here, each row corresponds to a spatial dimension of a mosquito trajectory at a specific time point, each column of $\boldsymbol{\Theta}$ corresponds to a mode of the behavioral forces in Eqs. (S2)-(S4), and all the coefficients in the basis-function expansion are grouped in a coefficient vector $\mathbf{w}$.

To perform Bayesian inference of $\mathbf{w}$, we minimize the negative log-posterior

$$-\ln P(\mathbf{w}|\{\mathbf{r}(t)\}) \sim -\ln P(\{\mathbf{r}(t)\}|\mathbf{w}) - \ln P(\mathbf{w}) \tag{S5}$$

with respect to $\mathbf{w}$ given the measurements $\{\mathbf{r}(t)\}$. To prevent overfitting, we follow previous work (*40, 41*) and impose a sparsity-promoting Gaussian prior over the coefficients

$$P(\mathbf{w}) = \prod_m \mathcal{N}(w_m|0,\gamma_m) = \prod_m (2\pi\gamma_m)^{-1/2} \exp\Big(-\frac{w_m^2}{2\gamma_m}\Big), \tag{S6}$$

where γ_m are hyperparameters representing the variances of the Gaussian distributions. Therefore, the negative log-prior becomes

$$-\ln P(\mathbf{w}) = \sum_m \frac{w_m^2}{2\gamma_m} + \sum_m \frac{1}{2}\ln(2\pi\gamma_m), \tag{S7}$$

which is similar to an L2 regularization on the coefficients $\mathbf{w}$ (*79, 80*). The negative log-likelihood function is thus given by

$$-\ln P(\{\mathbf{r}(t)\}|\mathbf{w}) = \frac{N}{2}\ln(2\pi) + \frac{1}{2}\ln|\mathbf{\Psi}| + \frac{1}{2}(\dot{\mathbf{v}} - \mathbf{\Theta}\mathbf{w})^T \mathbf{\Psi}^{-1}(\dot{\mathbf{v}} - \mathbf{\Theta}\mathbf{w}), \tag{S8}$$

where $\mathbf{\Psi} = \Delta \mathbf{I}_N$ is a diagonal matrix assuming Gaussian white noise, and N is the number of rows $\mathbf{\Theta}$ contains. Introducing Eqs. (S7, S8) into Eq. (S5), we obtain the posterior that follows a Gaussian distribution $P(\mathbf{w}|\{\mathbf{r}(t)\}) = \mathcal{N}(\mathbf{w};\boldsymbol{\mu},\mathbf{\Sigma})$, where the covariance matrix $\mathbf{\Sigma}$ and the mean $\boldsymbol{\mu}$ are given by

$$\mathbf{\Sigma} = (\mathbf{\Theta}^T \mathbf{\Psi}^{-1} \mathbf{\Theta}^T + \mathbf{\Gamma}^{-1})^{-1} \tag{S9}$$

$$\boldsymbol{\mu} = \mathbf{\Sigma}\mathbf{\Theta}^T \mathbf{\Psi}^{-1}\dot{\mathbf{v}}, \tag{S10}$$

and $\mathbf{\Gamma}$ is a diagonal matrix with $\mathbf{\Gamma}_{mm} = \gamma_m$.

To determine the values of hyperparameters in Eqs. (S9, S10), we employ a pragmatic procedure based on previous work (*81*), and choose γ_m and Δ to maximize the marginal likelihood $P(\{\mathbf{r}(t)\}|\Delta,\{\gamma_m\}) = \int P(\{\mathbf{r}(t)\}|\mathbf{w};\Delta)P(\mathbf{w}|\{\gamma_m\})d\mathbf{w}$. We use the Expectation Maximization (EM) method to iteratively update the values of γ_m and Δ. Specifically, given $\gamma_m^{(n)}$ and $\Delta^{(n)}$ from the previous iteration, we compute the current estimate of $\boldsymbol{\mu}^{(n)}$ and $\mathbf{\Sigma}^{(n)}$ using Eqs. (S9, S10). The EM approach gives the re-estimates

$$\gamma_m^{(n+1)} = \mathrm{E}_{\mathbf{w}\sim\mathcal{N}(\boldsymbol{\mu}^{(n)},\mathbf{\Sigma}^{(n)})}[\mathbf{w}_m^2] = (\boldsymbol{\mu}_m^{(n)})^2 + \mathbf{\Sigma}_{mm}^{(n)}), \text{ and} \tag{S11}$$

$$\Delta^{(n+1)} = \mathrm{E}_{\mathbf{w}\sim\mathcal{N}(\boldsymbol{\mu}^{(n)},\mathbf{\Sigma}^{(n)})}\Big[\frac{|\dot{\mathbf{v}} - \mathbf{\Theta}\mathbf{w}|^2}{N}\Big] = \frac{1}{N}\Big[|\dot{\mathbf{v}} - \mathbf{\Theta}\boldsymbol{\mu}^{(n)}|^2 + \Delta^{(n)}\sum_m (1 - \mathbf{\Sigma}_{mm}^{(n)}/\gamma_m^{(n)})\Big]. \tag{S12}$$

We note that when the degrees of freedom of the data samples N is much larger than the number of modes M, Eq. (S12) can be approximated by $\Delta^{(n+1)} \approx |\dot{\mathbf{v}} - \mathbf{\Theta}\boldsymbol{\mu}^{(n)}|^2/N$. Finally, we note that the scale parameters v_0 and d_0 in Eqs. (S2)-(S4) are chosen to minimize the residual $|\dot{\mathbf{v}} - \mathbf{\Theta}\boldsymbol{\mu}^{(n)}|^2/N$ through an additional layer of optimization outside of EM. Once the response to a specific target is learned, we can rescale the d_0 parameters to approximate the response to a larger or smaller target.

3.3 Model selection and verification

To generate models with varying complexities, we apply thresholding to the coefficients $\mathbf{w}$ and shrink the columns in $\mathbf{\Theta}$ sequentially. To identify the model that best balances the goodness of fit and model complexity, we use the negative Bayesian information criterion (BIC) (*82*), and select the model with the highest BIC score (see for example Fig. S2B). We note that the model dimensions, i.e., number of model parameters, selected by maximizing BIC is not invariant to the choice of basis, and therefore does not reflect the true complexity of the model. For example, a model represented by $a(x-b)^n$ has only two free parameters a and b, but requires $n+1$ coefficients when represented in the monomial basis. Maximizing BIC score within the monomial basis will therefore select the $n+1$-term polynomial model, since it is the smallest model in the candidate set capable of representing the true function exactly. In other words, BIC does not attempt to discover the intrinsic dimension of the underlying model; rather, it is a tool for choosing among a set of models under specific representations.

To verify the learned model for mosquito flight behaviors, we simulate Eq. (S1) with the learned behavioral forces and noises, and the experimental initial conditions. We compare the relevant statistics between the simulated and experimental trajectories. The stochastic differential equations are solved using Julia's DifferentialEquations.jl package (*83*) with the SOSRA solver.

3.4 Applications to synthetic data

Before applying our Bayesian inference framework to experimental data, we first test it on synthetic data of simulated mosquito trajectories. To mimic mosquitoes' free flight, we first simulate the Langevin equation Eq. (S1) with a speed potential force $\mathbf{f} = \alpha(v; v_0)\mathbf{v}$. Here, we choose a quadratic function for the rate $\alpha(v, v_0) = \beta(v_0^2 - v^2)$ where $\beta = 0.5$ s/m^2 is the prefactor and

$v_0 = 1$ m/s is the target flight speed. Indeed, simulations of this model yield trajectories that resemble the experimental data (see main Fig. 2), with a characteristic persistence length and moderate stochasticity in flight speed (Fig. S3A). We take these simulated trajectories as input data for the learning framework. The learned rate $\alpha(v; v_0)$ has a sparse representation with only a few nonzero coefficients and agrees quantitatively with the ground-truth function (Fig. S3B). Specifically, the Bayesian framework allows us to estimate the error of the learned $\alpha(v; v_0)$ as $\sigma_\alpha^2(v) = \boldsymbol{\theta}(v)^T \boldsymbol{\Sigma} \boldsymbol{\theta}(v)$ where $\boldsymbol{\Sigma}$ is the covariance matrix given by Eq. (S9) and $\boldsymbol{\theta} = [\theta_1(v), \theta_2(v), \ldots, \theta_m(v), \ldots]^T$ is a vector of basis functions. To further validate the learned model, we re-simulate Eq. (S1) with the learned $\mathbf{f}$ to generate new trajectories of mosquito flight (Fig. S3C), and compare their statistics with those of input data. Specifically, we examined the speed distribution, mean squared displacement, direction correlation, and speed correlation of the re-simulated trajectories, finding a close match with the input data (Fig. S3D–G).

Next, we test our Bayesian dynamical systems inference framework on a simulation model of sensory response. We consider a repulsive force $\mathbf{f} = \gamma \frac{v\hat{\mathbf{d}}}{1+\exp(\lambda(\hat{\mathbf{d}}\cdot\hat{\mathbf{v}}))}$ in response to a point source stimulus. Here, we choose $\gamma = 1$ and $\lambda = 5$. The simulated trajectories are shown in Fig. S4A. We use the basis-function expansion in Eq. (S4) and apply sparse Bayesian inference to learn the force magnitude. The close agreement between the learned force and the true force (Fig. S4B–D) demonstrates that our framework reliably estimates the behavioral force from time-series trajectories.

3.5 Comparison between experimental and simulation trajectory distributions

To quantify how closely the simulated mosquito trajectories match the experimental data (main Fig. 3 and 4), we computed a symmetric Kullback–Leibler (KL) distance between the corresponding trajectory distributions. Let $P(v, d, \hat{\mathbf{v}} \cdot \hat{\mathbf{d}})$ and $Q(v, d, \hat{\mathbf{v}} \cdot \hat{\mathbf{d}})$ be two distributions, where v denotes flight speed, d denotes the distance between the mosquito and target, and $\hat{\mathbf{v}} \cdot \hat{\mathbf{d}}$ describes flight direction. The symmetric KL distance D_{KL}^S is defined as:

$$D_{\mathrm{KL}}^S = \frac{1}{2}\left[D_{\mathrm{KL}}(P||Q) + D_{\mathrm{KL}}(Q||P)\right], \tag{S13}$$

where $D_{\mathrm{KL}}(P||Q)$ is the standard KL distance $D_{\mathrm{KL}}(P||Q) = \sum_{v,d,\hat{\mathbf{v}}\cdot\hat{\mathbf{d}}} P(v, d, \hat{\mathbf{v}} \cdot \hat{\mathbf{d}}) \ln \frac{P(v,d,\hat{\mathbf{v}}\cdot\hat{\mathbf{d}})}{Q(v,d,\hat{\mathbf{v}}\cdot\hat{\mathbf{d}})}$ (Fig. S10). This metric D_{KL}^S is non-negative and measures the "distance" between the two distribu-

tions.

The symmetric KL distances between the experimental and simulated trajectories are $D_{\text{KL}}^{S} = 0.20, 0.41$, and 0.31 for visual, CO_2, and combined visual and CO_2 cues, respectively. For reference, the corresponding distances between the experimental data and their best Gaussian fits are $D_{\text{KL}}^{S} = 0.36, 0.76$, and 0.98. This analysis demonstrates that the learned models capture the experimental distributions more accurately than simple Gaussian fits.

4 Interactive Website

To enable readers to interact with our learned mosquito behavioral models, we built an interactive web application. The application contains the learned models for no cue, visual cue, CO_2 cue, and the combined visual + CO_2 cues (Fig. S13). For visualization purposes on browser screens, the models are converted to 2D. The cue location in the model is defined at the center of image, which by default is an image of the MIT beaver mascot, but this can be changed through a user upload. The number of mosquitoes can also be changed by the user. The user can also move the location of the cue (the image) around and watch the mosquitoes respond.

The app works by running a Python server using Flask and a Julia server using Oxygen. Information about the user's interaction with the front end is sent to the Flask server, which updates the logic cue position and mosquito numbers. The Flask server then sends the positions and velocity data and the cue positions to the Julia server, which simulates the model for a specified amount of time. The Julia server solves the model with DifferentialEquations.jl and sends the updated positions for the next time period back to the Flask server and then to the front end visualization. The app is hosted on Railway, which deploys it from a Docker file in a linked GitHub repository.

Supplementary Figures

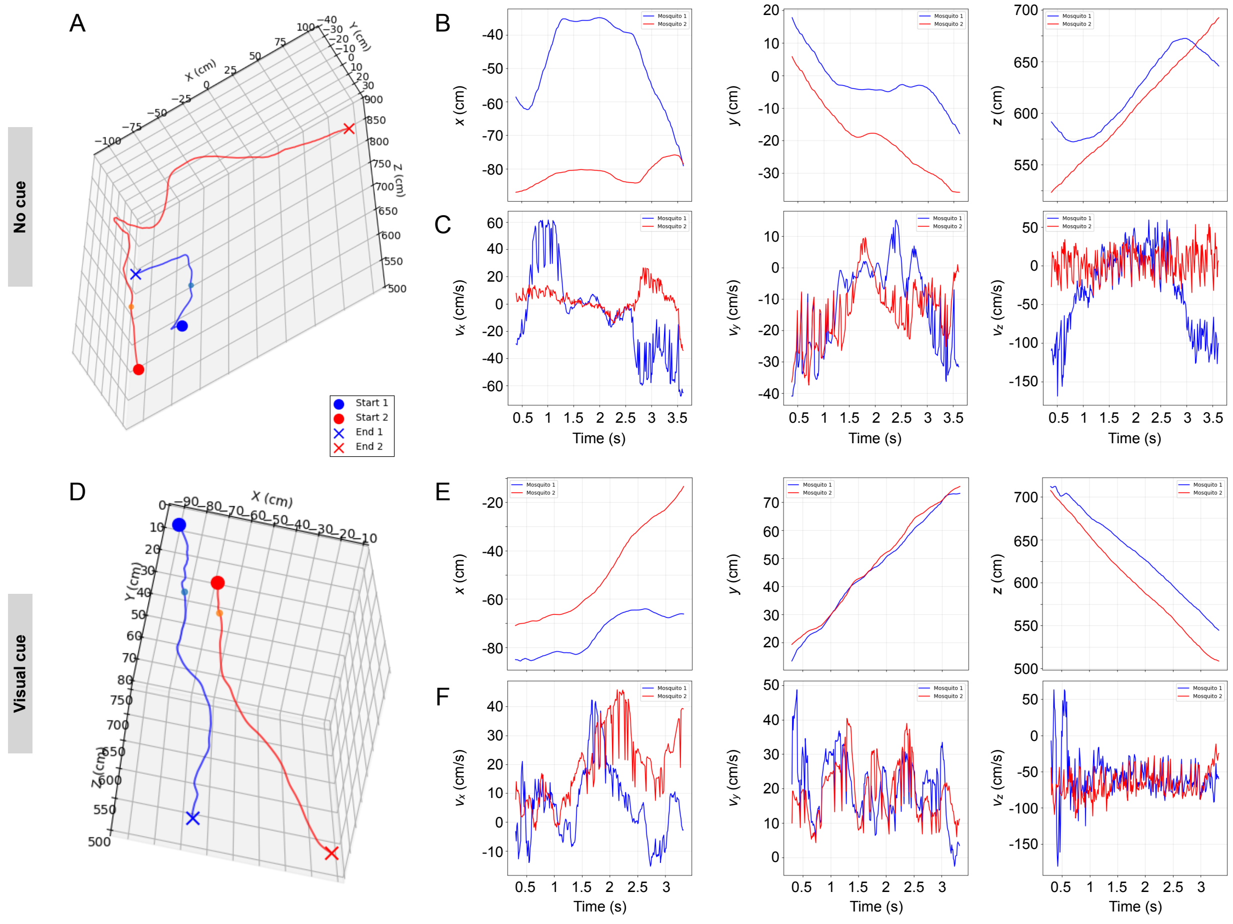

Fig. S1: **Example pair mosquito trajectories that follow similar paths for a brief period of time.** (**A–C**) Representative trajectory in the absence of sensory cues. (**A**) 3D reconstruction of the trajectories. Blue and red circles denote the starting positions of the two mosquitoes, respectively, and the cross markers indicate their end positions. (**B**) Time series of the x, y, and z positions. (**C**) Time series of the x, y, and z velocities. (**D–F**) Representative trajectory in the presence of a visual cue. Plots correspond to panels **A** – **C**.

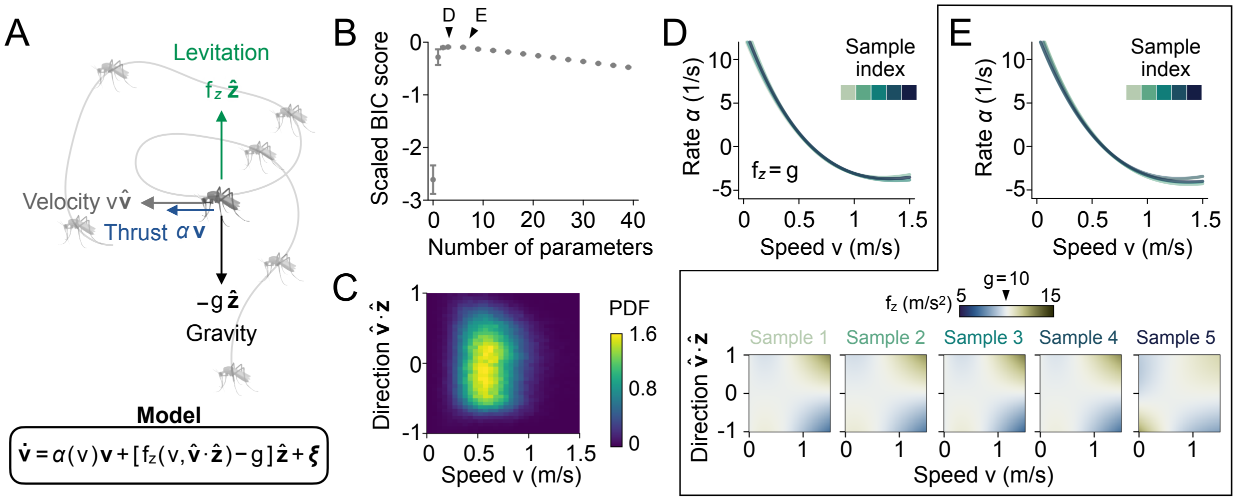

Fig. S2: **Bayesian dynamical systems learning of mosquito free-flight behaviors indicates the balance between levitation and gravity.** (**A**) Illustration of a mosquito flying with a velocity $\mathbf{v} = v\hat{\mathbf{v}}$. The (normalized) forces exerted on a mosquito include the thrust force $\alpha\mathbf{v}$, the levitational force $f_z\hat{\mathbf{z}}$, and the gravitational force $-g\hat{\mathbf{z}}$, where $g \approx 10$ m/s^2 is the gravitational acceleration constant. (**B**) Bayesian information criteria (BIC) scores for models with varying sparsity (number of model parameters). Arrowheads indicate two models shown in **D** and **E**. Error bars denote standard deviation based on 5 random subsamples, each containing 30 % of the total trajectories. (**C**) Probability density of recorded free flying mosquito trajectories at varying flight speed v and direction $\hat{\mathbf{v}} \cdot \hat{\mathbf{z}}$ where $\hat{\mathbf{v}} \cdot \hat{\mathbf{z}} = 1$ and -1 represent flying upward and downward, respectively. (**D**) The learned rate α of the model with the highest BIC score. This model has a constant levitational force $f_z = g$. Color scale indicates learning results for different subsamples. (**E**) The model with the second highest BIC score shows learned rates α highly similar to those in **D**, and levitational forces f_z approximately equal to g in the high data density region in **C**.

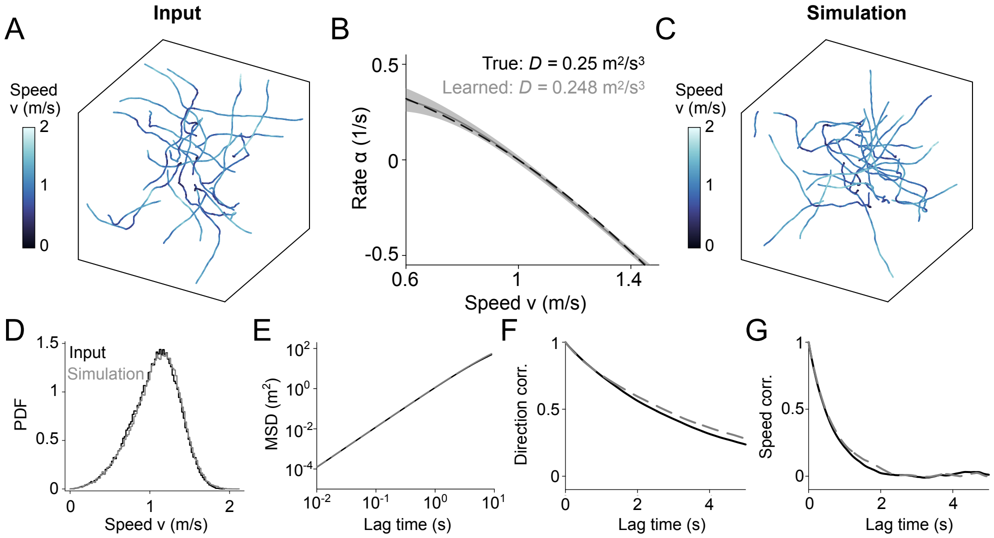

Fig. S3: **Bayesian inference framework accurately learns the Langevin equation with a speed potential force from synthetic data.** (**A**) Typical trajectories (20%) of the input synthetic data for the Bayesian inference framework. (**B**) The learned rate α (gray) accurately captures the ground truth (black). Shaded band indicates standard deviations computed from the posterior. See Sec. 3.4 for details. (**C**) Typical simulated trajectories (20%) of the learned model. (**D** – **G**) The simulation of the learned model and the input data show quantitative agreement in: (**D**) probability distribution of flight speed, (**E**) Mean squared displacement, (**F**) directional correlation, and (**G**) speed correlation.

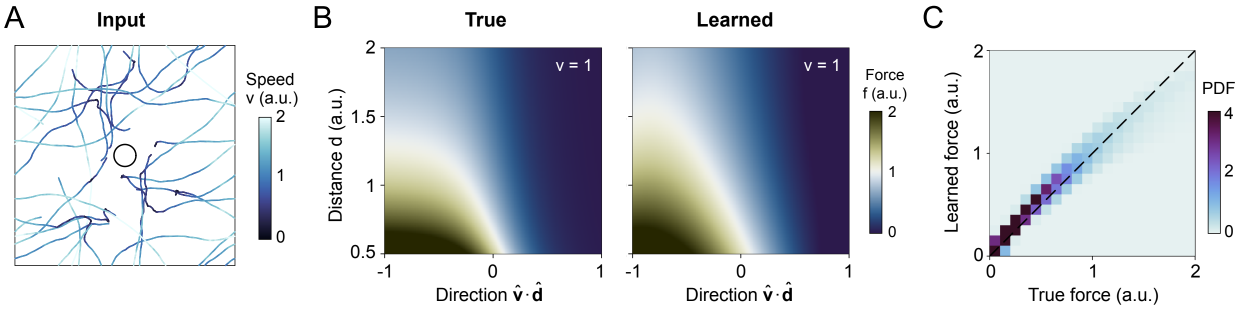

Fig. S4: **Bayesian inference framework accurately learns the response to external cues from synthetic data.** (**A**) Typical trajectories (10%) of the input synthetic data for the Bayesian inference framework. (**B**, **C**) The true and learned behavioral forces f for the specified speed $v = 1$. See Sec. 3.4 for details. (**C**) Density heatmap showing the true force versus the learned force for varying values of flight speed v, flight direction $\hat{\mathbf{v}} \cdot \hat{\mathbf{d}}$, and distance toward the target d. The dashed line indicates the diagonal $y = x$.

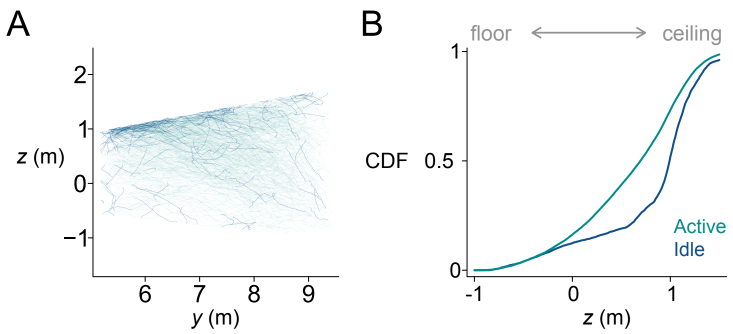

Fig. S5: **Mosquitoes are more likely to be idle than active when near the ceiling.** (**A**) Side-view trajectories of free-flying mosquitoes. The Idle state is colored blue and the active state is colored light green. (**B**) Cumulative probability distribution of the two states as a function of vertical coordinate z. Larger z values indicate being closer to the ceiling.

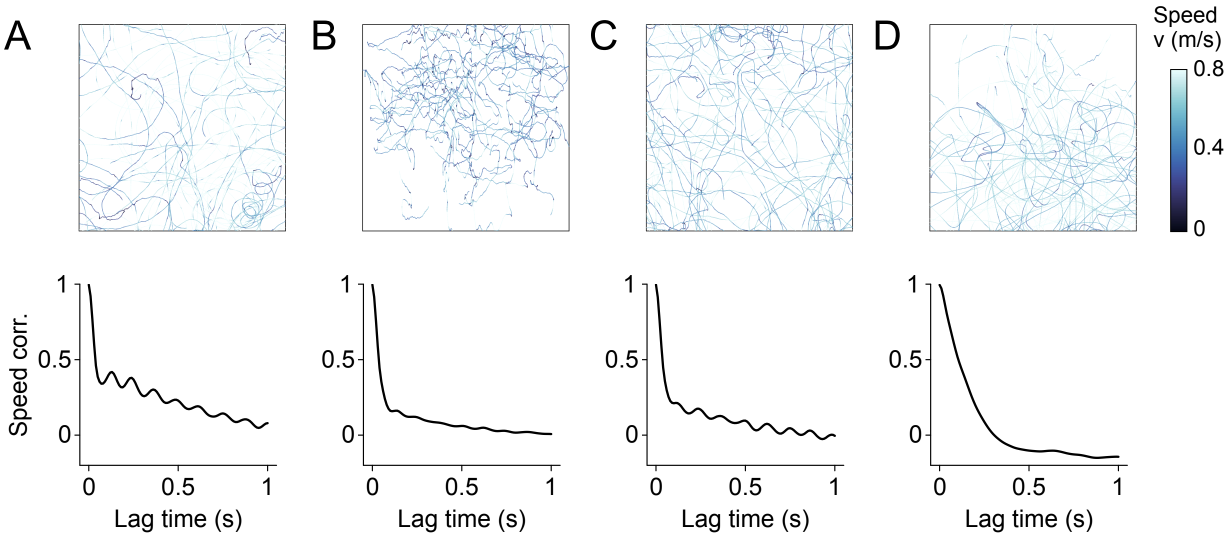

Fig. S6: **Speed oscillations in other insect species.** We obtained and analyzed trajectories for the following species and conditions: (**A**) *Anopheles stephensi* in a dark chamber without attractive cues, (**B**) *Anopheles stephensi* in a dark chamber with a bednet occupied by a male human, (**C**) *Anopheles stephensi* in a dark chamber with an unoccupied bednet, and (**D**) *Musca domestica* around a Zevo Flying Insect Trap from Joiner et al. (*84*), where the trials were conducted with headlights off. In **A**–**D**, the top sub-panels show 2D projections of 3D trajectories as in main Fig. 5**B**, and the bottom sub-panels show speed correlations as in main Fig. 2**J**.

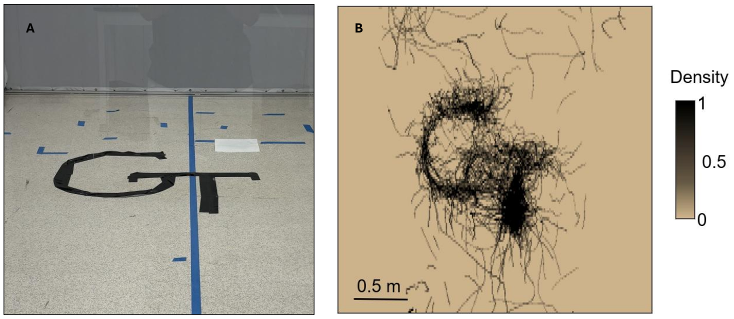

Fig. S7: **Mosquito trajectories form the GT Logo.** (**A**) The dark tape on the ground in the form of the GT Logo. (**B**) Mosquito trajectory concentration forming the Logo.

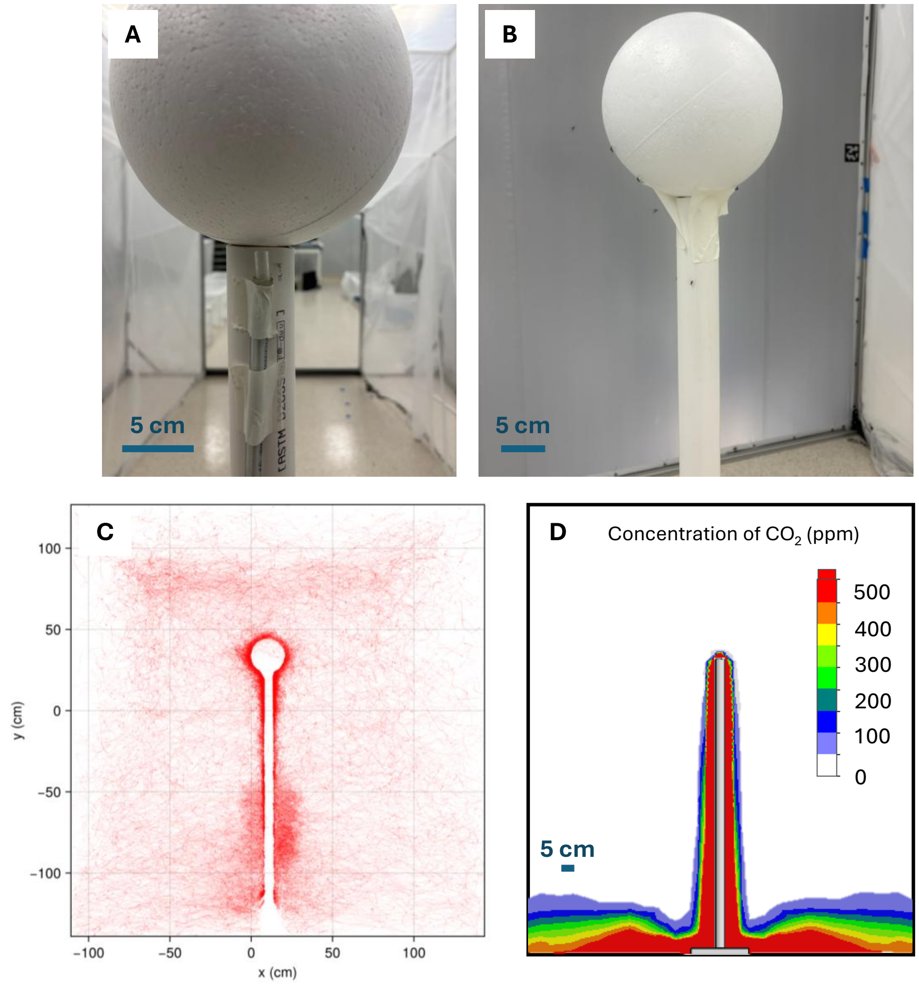

Fig. S8: **CO_2 experimental setup.** (**A**) Rear view of the setup for the experiment where the CO_2 is released into the chamber (**B**) Mosquitoes landing on the white sphere and stand (**C**) Trajectories of mosquitoes around the white sphere and stand (**D**) Solidworks simulation of the concentration of CO_2 above ambient air when released from the setup shown in **A**. Simulation is conducted without CO_2 release from the top of the stand. No sphere is included. Note mosquitoes can sense CO_2 concentrations as low as 103 ppm (*67*).

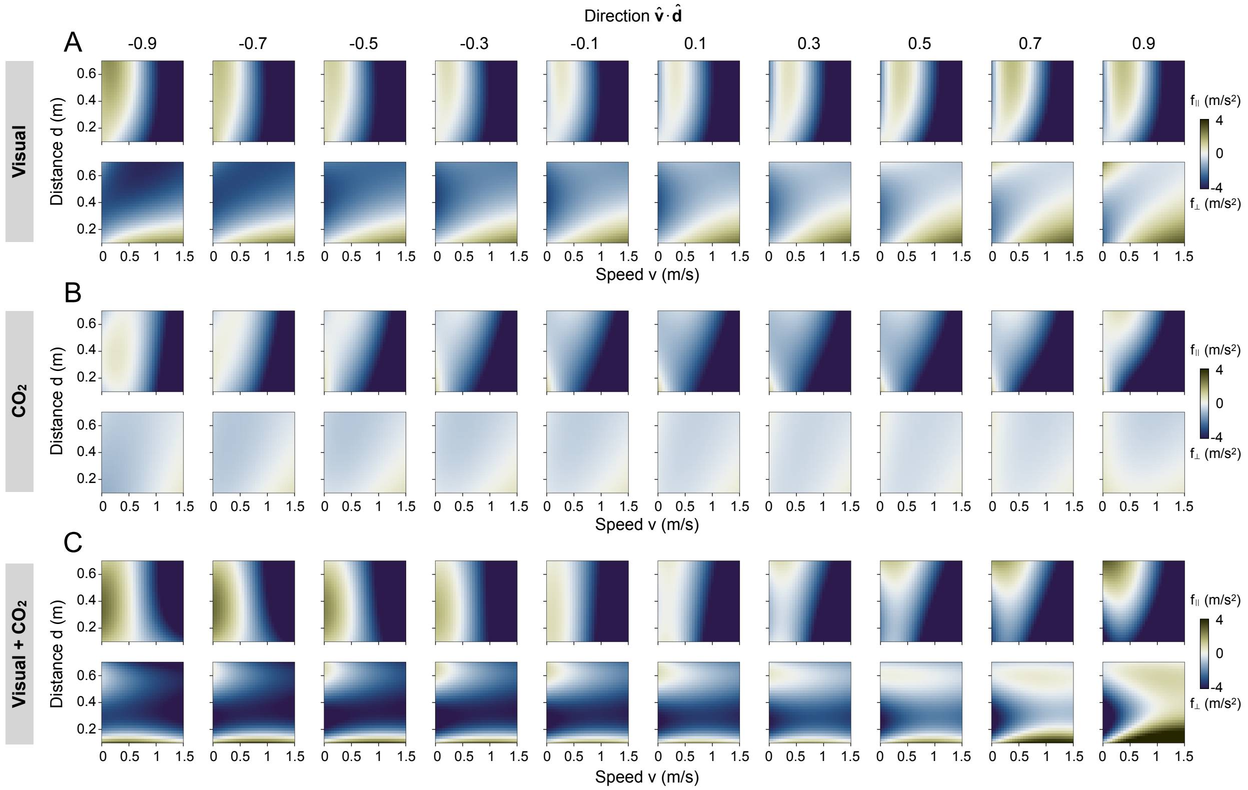

Fig. S9: **Learned behavioral forces demonstrate differential mosquito responses to various sensory cues.** (**A-C**) Learned behavioral forces acting parallel (*top*, $f_{\|}$) and perpendicular to (*bottom*, $f_{\perp}$) the mosquito flight direction, in response to (**A**) visual cues, (**B**) CO_2 cues, and (**C**) combined visual and CO_2 cues.

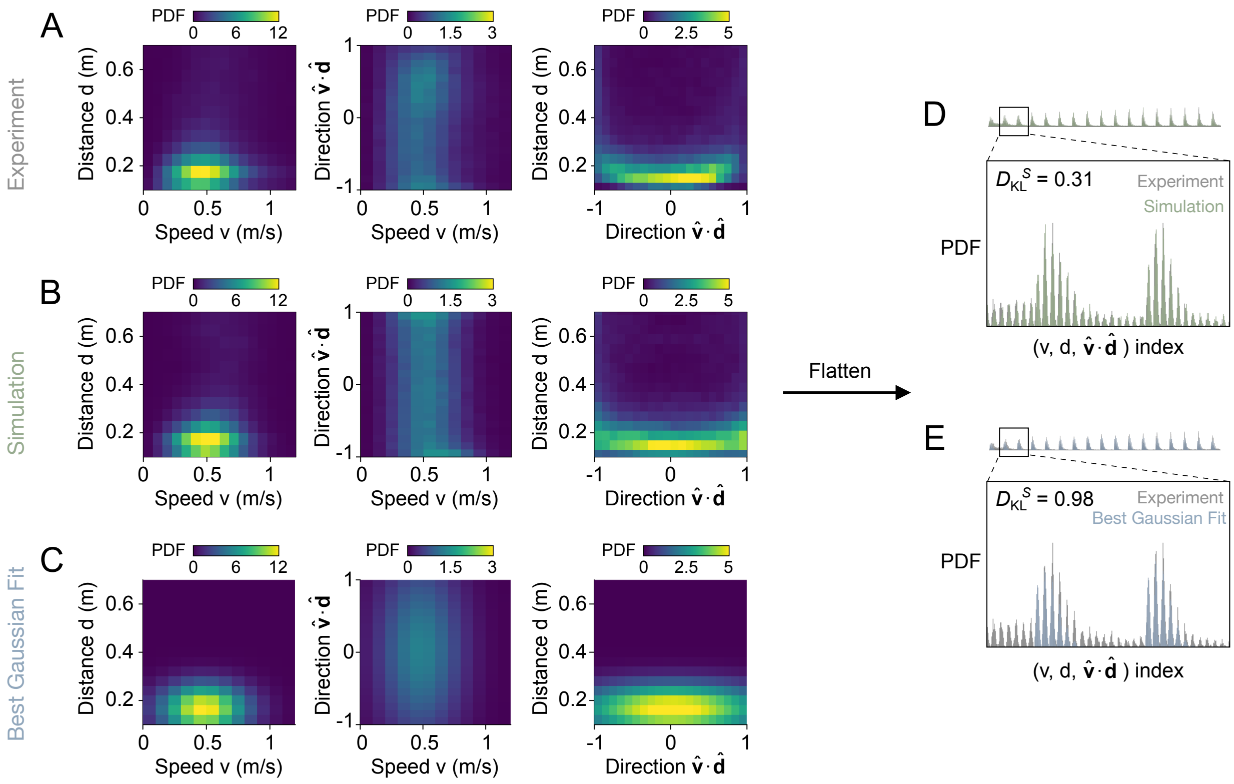

Fig. S10: **Quantifying the similarity between the experimental and simulation trajectory density distributions.** (**A**) Experimental and (**B**) simulation trajectory densities. Plots are the same as main Fig. 4**B** and **D**. (**C**) Best 3D Gaussian fit to the density in panel **A**. (**D**, **E**) The distributions shown in **A** – **C** are flattened into 1D distributions for direct visual comparison. We compute a symmetric KL divergence D_{KL}^{S} to quantify the dissimilarity between the distributions (see text). (**D**) An overlay of the experimental (gray bars) and simulation (green bars) distributions, with a D_{KL}^{S} of 0.31. (**E**) An overlay of the experimental distribution (gray bars) and its best Gaussian fit (blue bars), with a D_{KL}^{S} of 0.98. The lower D_{KL}^{S} value in **D** indicates that the simulation provides a better representation of the experimental data compared to a Gaussian fit.

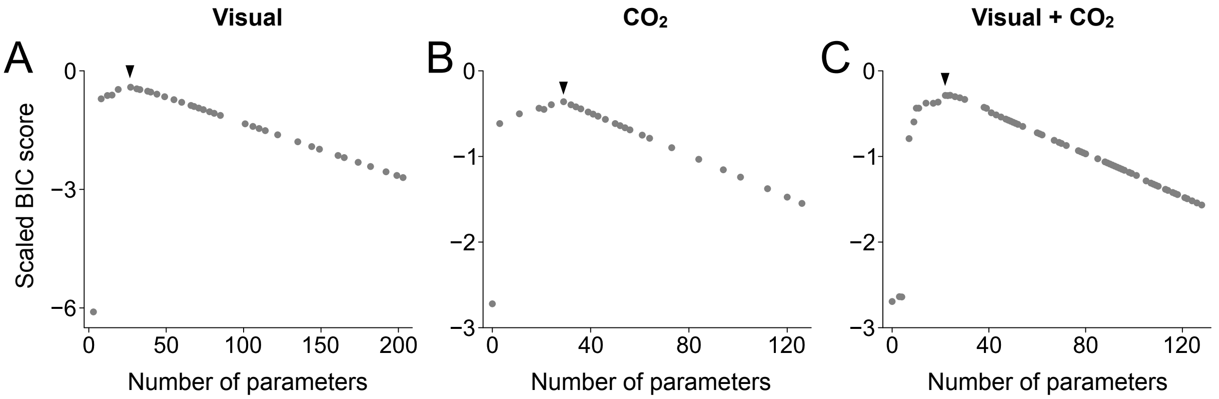

Fig. S11: **BIC scores for model selection.** BIC scores versus model parameter numbers for learned models of mosquito behavior in response to (**A**) visual, (**B**) CO_2, and (**C**) combined visual and CO_2 cues. The highest score (black arrowhead) indicates the optimal model, which balances goodness of fit with model complexity. For panels **A** – **C**, the optimal models have 27, 29, and 24 parameters, respectively.

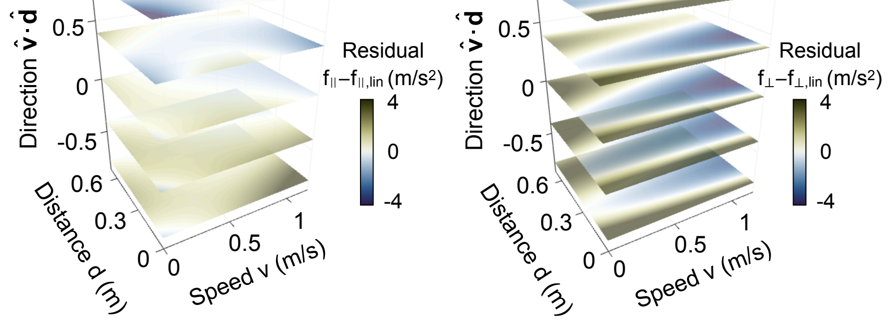

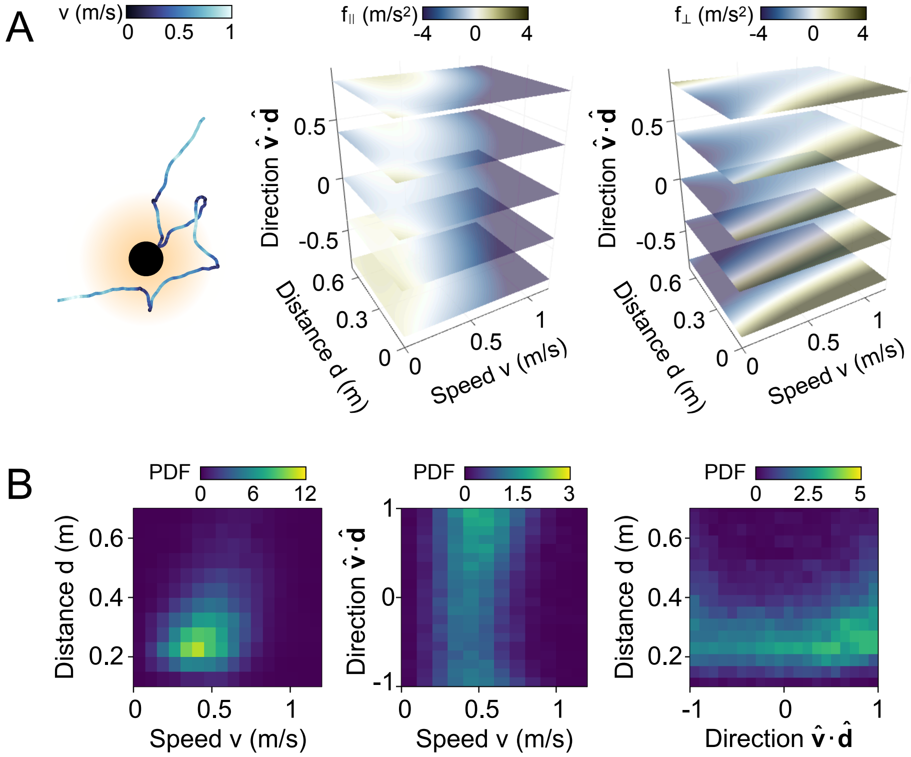

Fig. S12: **Mosquito response to combined visual and CO_2 cues is not a linear superposition of its response to individual cues.** (**A**) *Left*: A typical 2D simulation trajectory of the model using a linear superposition of uni-stimulus response (see main text for details). Heatmaps of the linearly reconstructed forces (*Middle*) $f_{||}$ and (*Right*) $f_{\perp}$. (**B**) 2D density maps of simulated mosquito trajectories using the behavioral forces in **A** fail to capture the experimental data in main Fig. 4**F**, indicating that the mosquito's response to combined visual and CO_2 cues is not a linear superposition of its response to individual cues.

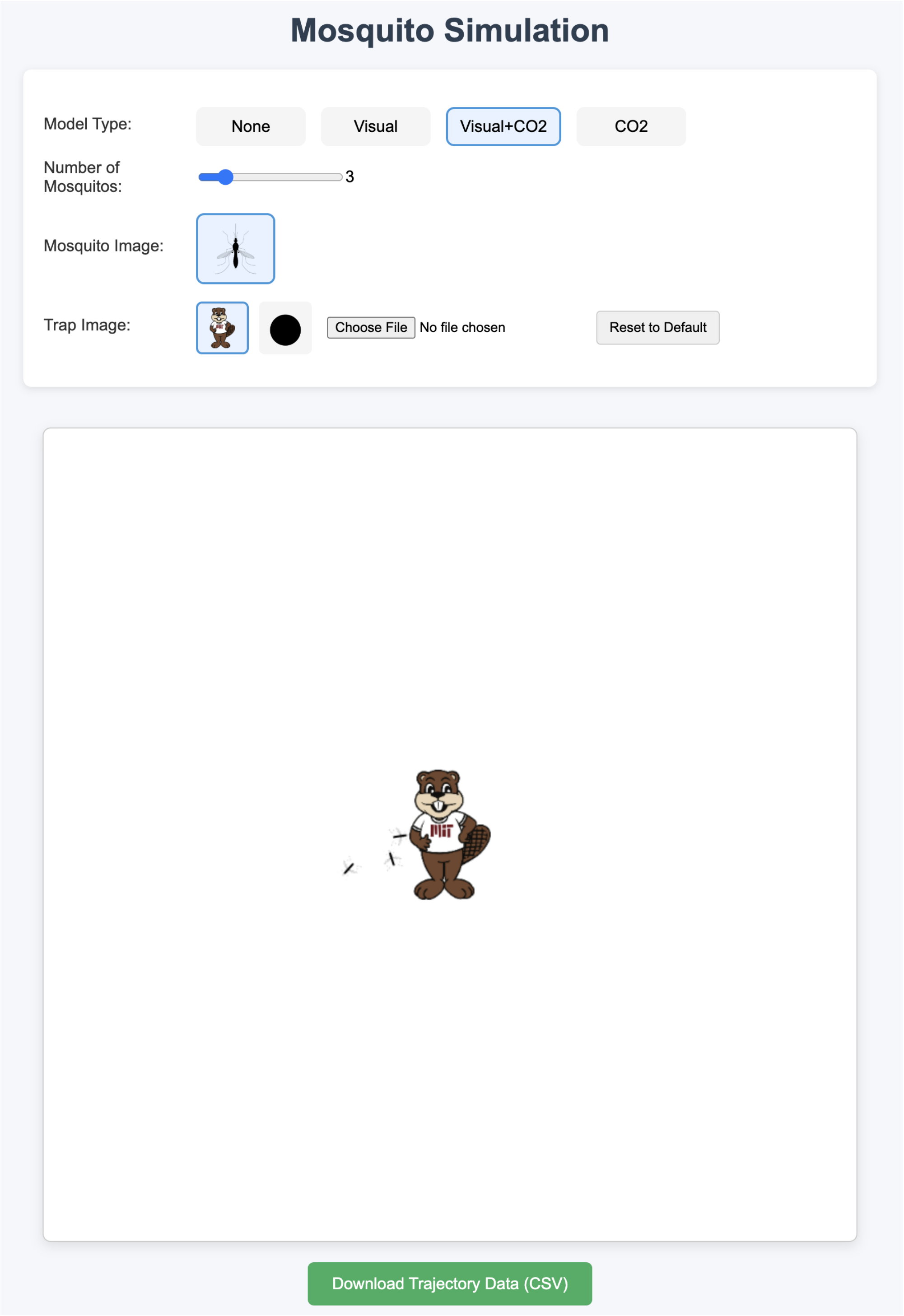

Fig. S13: **Screenshot of the interactive mosquito web application.** The figure shows the user interface used to configure the simulation of learned models, including options for model type, number of mosquitoes, and trap image.

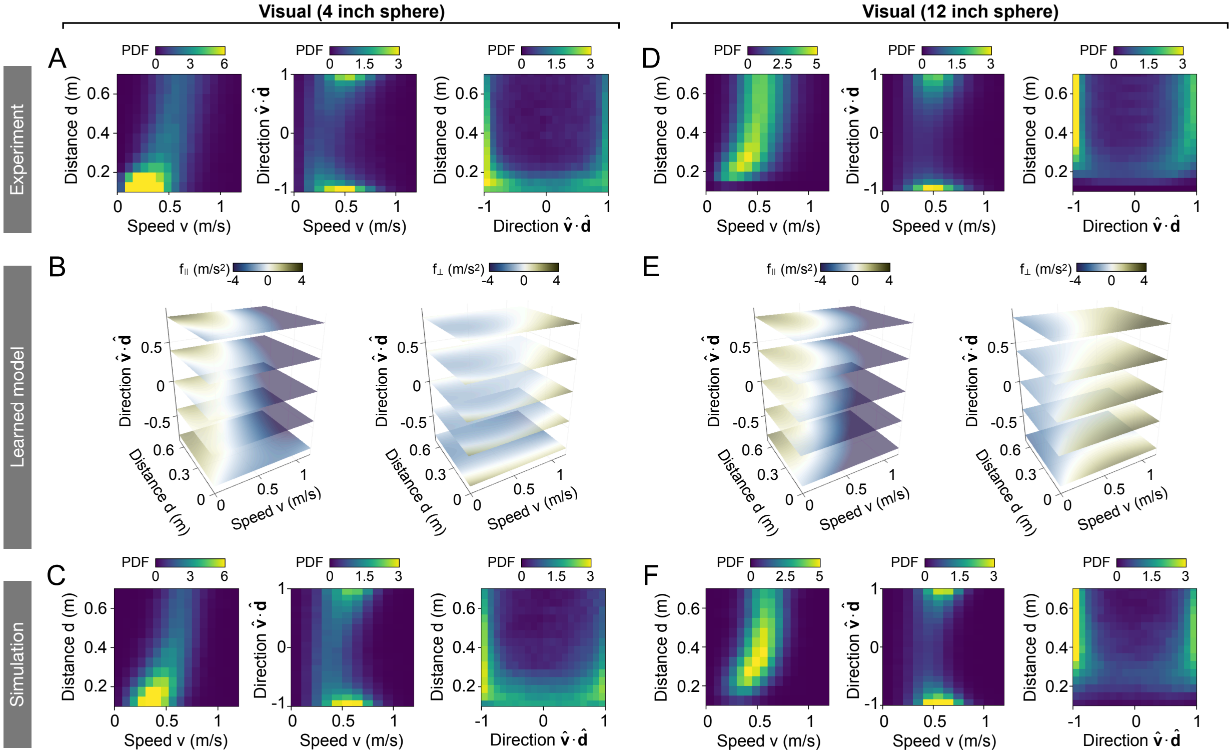

Fig. S14: **Mosquito responses to visual targets of varying sizes.** We apply the Bayesian inference framework to (**A**, **D**) the experimental data to learn (**B**, **E**) a dynamical model (see Sec. 3), and simulate the model to generate (**C**, **F**) synthetic trajectories. Results are shown for mosquito responses to black spheres with diameters of (**A**–**C**) 4 inches and (**D**–**F**) 12 inches. Figure panels correspond to main Fig. 3**C**–**E**.

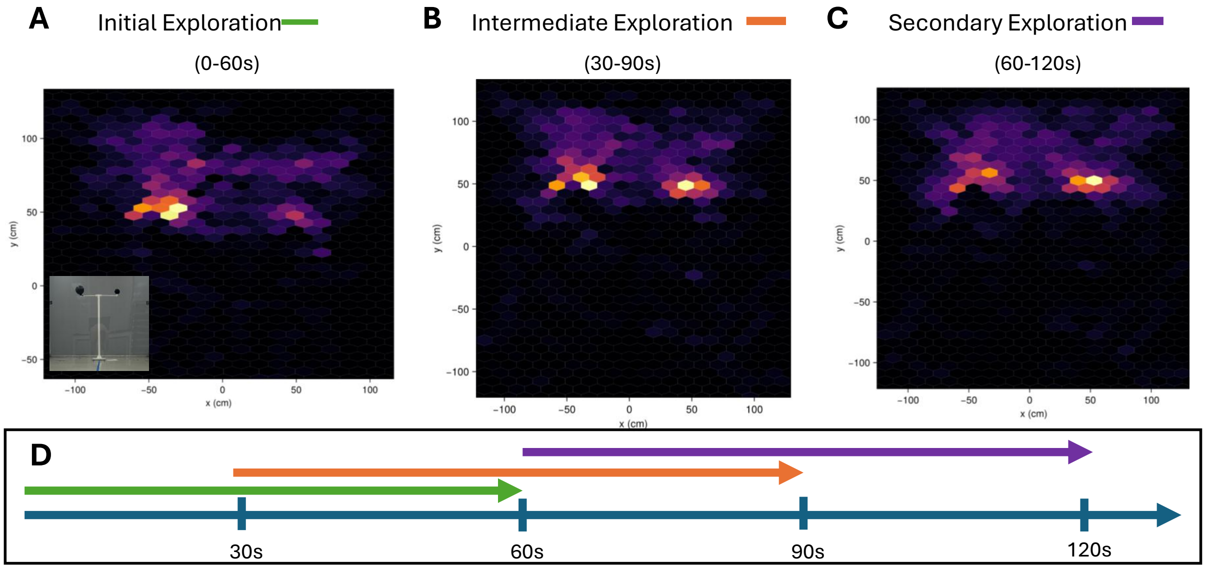

Fig. S15: **Timeline of mosquito trajectory distribution when exposed to two spheres.** (**A**) The heatmap shows the initial concentration around the larger sphere. Inset shows the setup of experiment, where the larger of the two black spheres (12in) is on the left and the smaller (4in) is on the right. (**B**) Heatmap showing mosquito densities moving towards the smaller sphere. (**C**) Heatmap showing mosquito densities concentrated around the smaller sphere. (**D**) Timeline of mosquito trajectory concentrations.

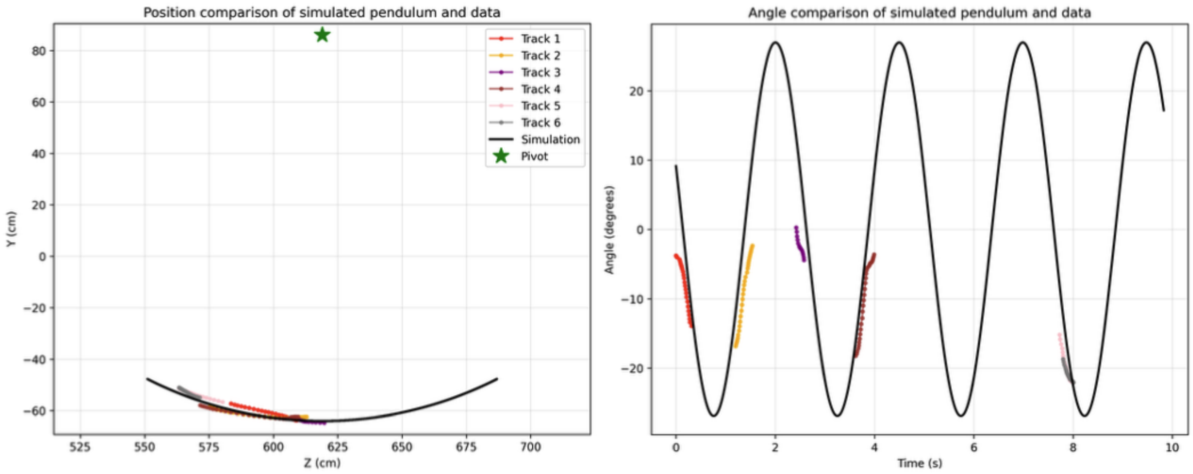

Fig. S16: **Pendulum experiment for data validation.** Using a simple pendulum constructed of 150 cm fishing wire and an M12 nut tied to the roof of the chamber. The nut is released at approximately 25 degrees and allowed to oscillate in the y-z plane. The trajectories are verified compared to the ideal pendulum shown in the figure.

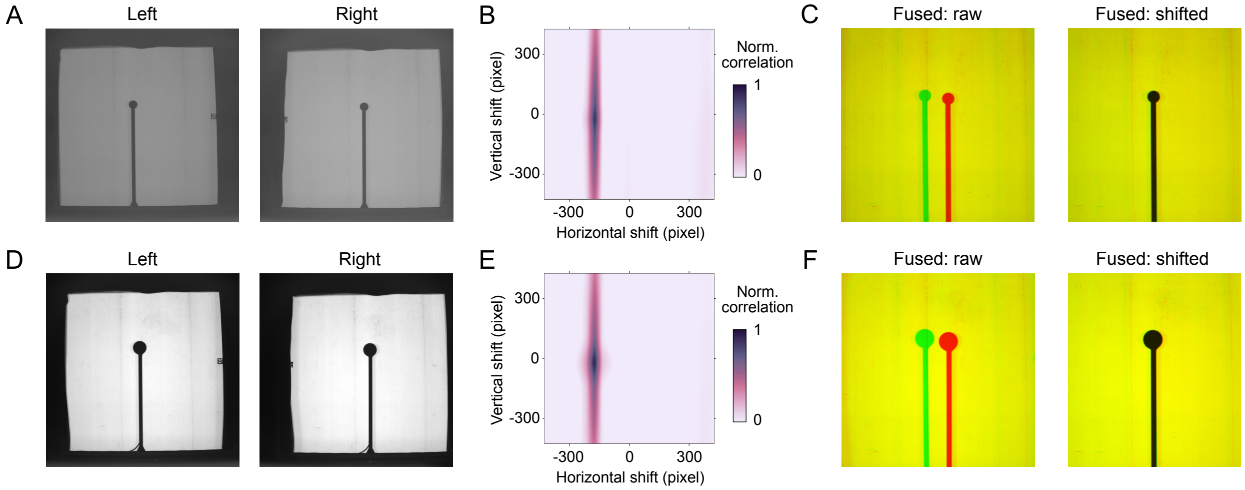

Fig. S17: **Stereo alignment and disparity estimation for depth accuracy analysis.** (**A**) Example grayscale images of a 4-inch sphere mounted on a pole, captured by the left and right cameras of the PFMD system. (**B**) Normalized cross-correlation map between the left and right images as a function of horizontal and vertical shifts. The cross-correlation shows a clear peak at a horizontal disparity of 171 pixels. (**C**) False-color overlays of the two views before (left) and after (right) applying the estimated disparity shift. The left view is mapped to the red channel, the right view to the green channel, and the blue channel is set to zero. The alignment after correction confirms the measured pixel disparity. (**D–F**) Disparity estimation using images of an 8-inch sphere mounted on a pole. Plots correspond to **A–C**. The estimated horizontal disparity is 174 pixels.

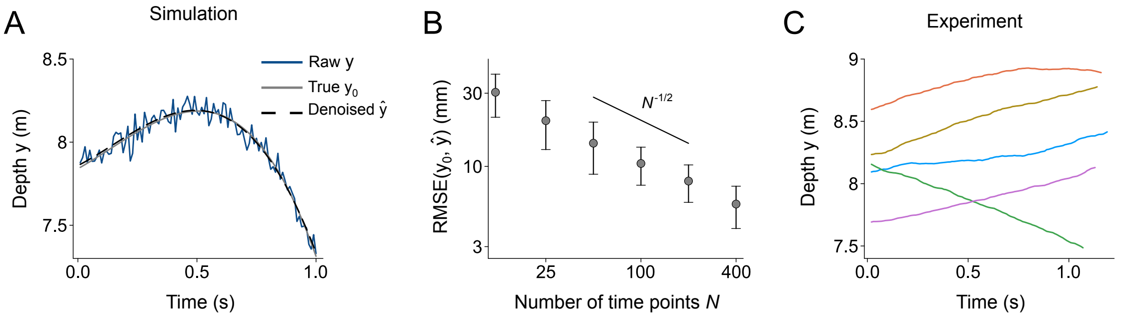

Fig. S18: **Denoising improves the depth tracking accuracy beyond the disparity-limited resolution.** (**A**) Simulated mosquito depth trajectory $y_0(t)$, contaminated with Gaussian white noise (σ = 50 mm) to mimic the optical depth error of the PFMD system ($y(t)$), and the corresponding denoised trajectory $\hat{y}(t)$. (**B**) Root mean square error (RMSE) between the denoised trajectory and the true trajectory versus the number of time points N, shown as mean ± s.d. over 100 replicates. The solid line indicates the expected scaling of $N^{-1/2}$. (**C**) Typical experimental trajectories showing smoother depth profiles than the synthetically noise-contaminated trace in **A**. The trajectories are selected randomly from the dataset analyzed in main Fig. 2, specifically those filtered to have a duration of roughly 1 s and a depth around 8 m.

Supplementary Tables

	species	# of track data points	# of trajectories	time (min)	# of data points per minute	# of trajectories per minute	# of insects at a time	dimension of measurement
Kennedy (1940)	Mosquito (*Ae. aegypti*)	-	-	5	-	-	10	no
Geier et al. (1999)	Mosquito (*Ae. aegypti*)	-	-	20	-	-	20	no
Spitzen et al. (2013)	Mosquito (*An. gambiae*)	15,000	1	10	1,500	0.1	1	3D
Hawkes and Gibson (2016)	Mosquito (*An. coluzzii*)	-	69	210	-	0.3	5	3D
Cribellier et al. (2018)	Mosquito (*An. coluzzii*)	-	2,570	795	-	3.2	10	3D
Sinhuber et al. (2019)	Midge (*Ch. riparius*)	600,000	5,242	3	240,000	2,096.8	>100	3D
Cribellier et al. (2020)	Mosquito (*An. coluzzii*)	-	13,618	1,105	-	12.3	50	3D
Amos et al. (2020)	Mosquito (*Ae. aegypti*)	-	718	5,220	-	0.1	100	2D
Amos et al. (2020)	Mosquito (*Ae. aegypti*)	-	334	4,920	-	0.1	100	2D
Hinze et al. (2021)	Mosquito (*An. gambiae*)	-	-	10	-	-	1	3D
Breugel et al. (2015)	Mosquito (*Ae. aegypti*)	-	3,602	180	-	20.0	120	3D
Alonso et al. (2022)	Mosquito (*Ae. aegypti*)	-	5,575	180	-	31.0	50	3D
Our study	Mosquito (*Ae. aegypti*)	3,361,595	28,400	25	134,464	1,136.0	100	3D

Table S1: **Table of experimental metrics comparing previous studies mapping insect trajectories in 3D.** Our study values are derived from the "Black 4-inch Sphere" experiment.

Date:	Experiment Name:	Duration:	Number of Mosquitoes:	Number of Trajectories	Number of Data Points	File Name
1/31/2025	White Body Black Head	10	50	7655	327994	1-31-25-White-Body-Black-Head.csv
1/31/2025	Anopheles	10	50	4123	181097	1-31-25-Anopheles.csv
11/22/2024	Half White Half Black Body	10	50	9467	392857	11-22-24-Half-White-Half-Black-Body.csv
11/22/2024	White 8in CO2 Black 8in No CO2	30	30	19434	1321771	11-22-24-White-8in-CO2-vs-Black-8in-No-CO2.csv
11/22/2024	Black 8in CO2	20	50	20522	1589323	11-22-24-Black-8in-CO2.csv
11/22/2024	White 8in CO2	20	50	22404	1379476	11-22-24-White-8in-CO2.csv
5/3/2024	Black 4in Sphere	25	50	20791	1778418	5-3-24-Black-Sphere-Unknown-Size.csv
5/3/2024	Black 4in Sphere CO2	25	50	36644	3059827	5-3-24-Black-Sphere-CO2-Unknown-Size.csv
5/3/2024	White 4in Sphere	25	50	14616	1397147	5-3-24-White-Sphere-Unknown-Size.csv
6/21/2024	Black 12in Sphere	25	50	34084	3762295	6-21-24-Black-12in-Sphere.csv
6/21/2024	Black 16in Sphere	25	50	21814	2149102	6-21-24-Black-16in-Sphere.csv
6/21/2024	Black 4 in Sphere	25	100	28400	3361595	6-21-24-Black-4in-Sphere.csv
6/21/2024	Black 4in Sphere vs Black 16in Sphere	25	50	12678	1118999	6-21-24-Black-4in-Sphere-vs-Black-16in-Sphere.csv
6/21/2024	Black 4in Sphere vs Black 12in Sphere	25	50	16007	1373072	6-21-24-Black-4in-Sphere-vs-Black-12in-Sphere.csv
7/22/2024	Black 4in Sphere vs Black 16in Sphere	20	50	25661	2587371	7-22-24-Black-4in-Sphere-vs-Black-16in-Sphere.csv
7/22/2024	Black 4in Sphere vs Black 4in Sphere	20	50	22022	2217765	7-22-24-Black-4in-Sphere-vs-Black-4in-Sphere.csv
7/22/2024	Black 4in Sphere vs Black 8in Sphere	20	50	15438	1583819	7-22-24-Black-4in-Sphere-vs-Black-8in-Sphere.csv
7/22/2024	Inverted Black 4in Sphere vs Black 12in Sphere	20	50	31435	2932778	7-22-24-Inverted-Black-4in-Sphere-vs-Black-12in-Sphere.csv
10/27/2023	100 Mosquitoes Empty Chamber	20	100	11963	1752329	10-27-23-100-Mosquitoes-Empty-Chamber.csv
5/1/2023	Person Regular Clothes	10	50	8090	362835	5-1-23-Person-Regular-Clothes.csv
1/31/2025	GT Logo	15	100	93972	7039925	1-31-25-GT-Logo.csv

Table S2: **Table of detailed metrics for all experimental trials.** The table lists the specific parameters and corresponding results for each trial conducted, including duration, number of mosquitoes, trajectories, and total data points recorded.

Caption for Supplementary Movies

- Movie S1: 3D experimental trajectories of mosquitoes around a human target, overlayed with Polycam 3D scan of the human target
- Movie S2: 3D experimental trajectories of mosquitoes around a human target wearing a half black and half white “Janus outfit” .
- Movie S3: Experimental trajectories of mosquitoes around an 8-inch (0.1 m) black sphere, acting as a visual cue.
- Movie S4: Experimental trajectories of mosquitoes around a white sphere with CO_2 being released at 0.24 L/min (the CO_2 levels of human breathing) underneath the sphere.
- Movie S5: Simulated trajectories of mosquitoes around a black sphere (visual target).
- Movie S6: Simulated trajectories of mosquitoes around an invisible target releasing CO_2.
- Movie S7: Experimental trajectories of mosquitoes responding to a target with both visual and CO_2 cues.
- Movie S8: Simulated trajectories of mosquitoes around a target with both visual and CO_2 cues.

REFERENCES

1. O. Omodior, M. C. Luetke, E. J. Nelson, Mosquito-borne infectious disease, risk-perceptions, and personal protective behavior among US international travelers. *Prev. Med. Rep.* **12**, 336–342 (2018).

2. J. Wang, Z. Zhu, Novel paradigm of mosquito-borne disease control based on self-powered strategy. *Front. Public Health* **11**, 1115000 (2023).

3. C. S. McBride, F. Baier, A. B. Omondi, S. A. Spitzer, J. Lutomiah, R. Sang, R. Ignell, L. B. Vosshall, Evolution of mosquito preference for humans linked to an odorant receptor. *Nature* **515**, 222–227 (2014).

4. D. A. Yee, C. D. Bermond, L. J. Reyes-Torres, N. S. Fijman, N. A. Scavo, J. Nelsen, S. H. Yee, Robust network stability of mosquitoes and human pathogens of medical importance. *Parasit. Vectors* **15**, 216 (2022).

5. A. Hinze, S. Hill, R. Ignell, "Chapter 9: Odour-mediated host selection and discrimination in mosquitoes," in *Sensory Ecology of Disease Vectors* (Wageningen Academic Publishers, 2022), pp. 253–276.

6. F. Van Breugel, J. Riffell, A. Fairhall, M. H. Dickinson, Mosquitoes use vision to associate odor plumes with thermal targets. *Curr. Biol.* **25**, 2123–2129 (2015).

7. T. D. De, R. Dixit, "Neuro-olfactory regulation and salivary actions: A coordinated event for successful blood-feeding behavior of mosquitoes," in *Sino-Nasal and Olfactory System Disorders* (IntechOpen, 2020), pp. 73–95.

8. A. Cribellier, J. A. van Erp, A. Hiscox, M. J. Lankheet, J. L. van Leeuwen, J. Spitzen, F. T. Muijres, Flight behaviour of malaria mosquitoes around odour-baited traps: Capture and escape dynamics. *R. Soc. Open Sci.* **5**, 180246 (2018).

9. M. Z. Liu, L. B. Vosshall, General visual and contingent thermal cues interact to elicit attraction in female *Aedes aegypti* mosquitoes. *Curr. Biol.* **29**, 2250–2257 (2019).

10. E. Warrant, M. Dacke, Vision and visual navigation in nocturnal insects. *Annu. Rev. Entomol.* **56**, 239–254 (2011).

11. D. Alonso San Alberto, C. Rusch, Y. Zhan, A. D. Straw, C. Montell, J. A. Riffell, The olfactory gating of visual preferences to human skin and visible spectra in mosquitoes. *Nat. Commun.* **13**, 555 (2022).

12. M. Gillies, T. Wilkes, Field experiments with a wind tunnel on the flight speed of some West African mosquitoes (Diptera: Culicidae). *Bull. Entomol. Res.* **71**, 65–70 (1981).

13. M. Gillies, T. Wilkes, Evidence for downwind flights by host-seeking mosquitoes. *Nature* **252**, 388–389 (1974).

14. B. D. Sumner, R. T. Cardé, Primacy of human odors over visual and heat cues in inducing landing in female *Aedes aegypti* mosquitoes. *J. Insect Behav.* **35**, 31–43 (2022).

15. M. F. Cooperband, R. T. Cardé, Comparison of plume structures of carbon dioxide emitted from different mosquito traps. *Med. Vet. Entomol.* **20**, 1–10 (2006).

16. C. J. McMeniman, R. A. Corfas, B. J. Matthews, S. A. Ritchie, L. B. Vosshall, Multimodal integration of carbon dioxide and other sensory cues drives mosquito attraction to humans. *Cell* **156**, 1060–1071 (2014).

17. T. Dekker, M. Geier, R. T. Cardé, Carbon dioxide instantly sensitizes female yellow fever mosquitoes to human skin odours. *J. Exp. Biol.* **208**, 2963–2972 (2005).

18. E. Barredo, J. I. Raji, M. Ramon, M. DeGennaro, J. Theobald, Carbon dioxide and blood-feeding shift visual cue tracking during navigation in *Aedes aegypti* mosquitoes. *Biol. Lett.* **18**, 20220270 (2022).

19. W. J. Laursen, G. Budelli, R. Tang, E. C. Chang, R. Busby, S. Shankar, R. Gerber, C. Greppi, R. Albuquerque, P. A. Garrity, Humidity sensors that alert mosquitoes to nearby hosts and egg-laying sites. *Neuron* **111**, 874–887 (2023).

20. V. Choumet, T. Attout, L. Chartier, H. Khun, J. Sautereau, A. Robbe-Vincent, P. Brey, M. Huerre, O. Bain, Visualizing non infectious and infectious *Anopheles gambiae* blood feedings in naive and saliva-immunized mice. *PLOS ONE* **7**, e50464 (2012).

21. B. A. Amos, K. M. Staunton, S. A. Ritchie, R. T. Cardé, Attraction versus capture: Efficiency of BG-Sentinel trap under semi-field conditions and characterizing response behaviors for female *Aedes aegypti* (Diptera: Culicidae). *J. Med. Entomol.* **57**, 884–892 (2020).

22. M. F. Cooperband, R. T. Cardé, Orientation of *Culex* mosquitoes to carbon dioxide-baited traps: Flight manoeuvres and trapping efficiency. *Med. Vet. Entomol.* **20**, 11–26 (2006).

23. E. Jatta, M. Carrasco-Tenezaca, M. Jawara, J. Bradley, S. Ceesay, U. D'Alessandro, D. Jeffries, B. Kandeh, D. S.-H. Lee, M. Pinder, A. L. Wilson, J. Knudsen, S. W. Lindsay, Impact of increased ventilation on indoor temperature and malaria mosquito density: An experimental study in The Gambia. *J. R. Soc. Interface* **18**, 20201030 (2021).

24. S. Majeed, S. R. Hill, T. Dekker, R. Ignell, Detection and perception of generic host volatiles by mosquitoes: Responses to CO_2 constrains host-seeking behaviour. *R. Soc. Open Sci.* **4**, 170189 (2017).

25. R. de Jong, B. G. Knols, "Selection of biting sites by mosquitoes," in *Ciba Foundation Symposium 200: Olfaction in Mosquito-Host Interactions* (Wiley Online Library, 2007), pp. 89–108.

26. T. Dekker, W. Takken, B. G. J. Knols, E. Bouman, S. van de Laak, A. de Bever, P. W. T. Huisman, Selection of biting sites on a human host by *Anopheles gambiae* ss, *An. arabiensis* and *An. quadriannulatus*. *Entomol. Exp. Appl.* **87**, 295–300 (1998).

27. F. Hawkes, G. Gibson, Seeing is believing: The nocturnal malarial mosquito *Anopheles coluzzii* responds to visual host-cues when odour indicates a host is nearby. *Parasit. Vectors* **9**, 320 (2016).

28. M. Sinhuber, K. van der Vaart, R. Ni, J. G. Puckett, D. H. Kelley, N. T. Ouellette, Three-dimensional time-resolved trajectories from laboratory insect swarms. *Sci. Data* **6**, 190036 (2019).

29. J. Spitzen, C. W. Spoor, F. Grieco, C. ter Braak, J. Beeuwkes, S. P. van Brugge, S. Kranenbarg, L. P. J. J. Noldus, J. L. van Leeuwen, W. Takken, A 3D analysis of flight behavior of *Anopheles gambiae sensu stricto* malaria mosquitoes in response to human odor and heat. *PLOS ONE* **8**, e62995 (2013).

30. T. Dekker, R. T. Cardé, Moment-to-moment flight manoeuvres of the female yellow fever mosquito (*Aedes aegypti* L.) in response to plumes of carbon dioxide and human skin odour. *J. Exp. Biol.* **214**, 3480–3494 (2011).

31. L. E. Muir, M. J. Thorne, B. H. Kay, *Aedes aegypti* (Diptera: Culicidae) vision: Spectral sensitivity and other perceptual parameters of the female eye. *J. Med. Entomol.* **29**, 278–281 (1992).

32. L. E. Muir, B. H. Kay, M. J. Thorne, *Aedes aegypti* (Diptera: Culicidae) vision: Response to stimuli from the optical environment. *J. Med. Entomol.* **29**, 445–450 (1992).

33. F. Hawkes, J. Zeil, G. Gibson, "Chapter 19: Vision in mosquitoes, in *Sensory Ecology of Disease Vectors*" (Wageningen Academic Publishers, 2022), pp. 511–533.

34. N. J. Mlot, C. A. Tovey, D. L. Hu, Fire ants self-assemble into waterproof rafts to survive floods. *Proc. Natl. Acad. Sci. U.S.A.* **108**, 7669–7673 (2011).

35. O. Peleg, J. M. Peters, M. K. Salcedo, L. Mahadevan, Collective mechanical adaptation of honeybee swarms. *Nat. Phys.* **14**, 1193–1198 (2018).

36. G. J. Berman, D. M. Choi, W. Bialek, J. W. Shaevitz, Mapping the stereotyped behaviour of freely moving fruit flies. *J. R. Soc. Interface* **11**, 20140672 (2014).

37. J. F. Méndez-Valderrama, Y. A. Kinkhabwala, J. Silver, I. Cohen, T. Arias, Density-functional fluctuation theory of crowds. *Nat. Commun.* **9**, 3538 (2018).

38. A. Attanasi, A. Cavagna, L. D. Castello, I. Giardina, T. S. Grigera, A. Jelić, S. Melillo, L. Parisi, O. Pohl, E. Shen, M. Viale, Information transfer and behavioural inertia in starling flocks. *Nat. Phys.* **10**, 691–696 (2014).

39. K. Bozek, L. Hebert, Y. Portugal, A. S. Mikheyev, G. J. Stephens, Markerless tracking of an entire honey bee colony. *Nat. Commun.* **12**, 1733 (2021).

40. M. E. Tipping, Sparse Bayesian learning and the relevance vector machine. *J. Mach. Learn. Res.* **1**, 211–244 (2001).

41. D. P. Wipf, B. D. Rao, Sparse Bayesian learning for basis selection. *IEEE Trans. Signal. Process.* **52**, 2153–2164 (2004).

42. W. Pan, Y. Yuan, J. Gonçalves, G.-B. Stan, A sparse Bayesian approach to the identification of nonlinear state-space systems. *IEEE Trans Automat Contr* **61**, 182–187 (2015).

43. Y. Yuan, X. Tang, W. Zhou, W. Pan, X. Li, H.-T. Zhang, H. Ding, J. Goncalves, Data driven discovery of cyber physical systems. *Nat. Commun.* **10**, 4894 (2019).

44. R. Fuentes, R. Nayek, P. Gardner, N. Dervilis, T. Rogers, K. Worden, E. J. Cross, Equation discovery for nonlinear dynamical systems: A Bayesian viewpoint. *Mech. Syst. Signal Process.* **154**, 107528 (2021).

45. Y. Huang, Y. Mabrouk, G. Gompper, B. Sabass, Sparse inference and active learning of stochastic differential equations from data. *Sci. Rep.* **12**, 21691 (2022).

46. S. M. Hirsh, D. A. Barajas-Solano, J. N. Kutz, Sparsifying priors for Bayesian uncertainty quantification in model discovery. *R. Soc. Open Sci.* **9**, 211823 (2022).

47. N. M. Mangan, S. L. Brunton, J. L. Proctor, J. N. Kutz, Inferring biological networks by sparse identification of nonlinear dynamics. *IEEE Trans. Mol. Biol. Multi Scale Commun.* **2**, 52–63 (2016).

48. D. B. Brückner, P. Ronceray, C. P. Broedersz, Inferring the dynamics of underdamped stochastic systems. *Phys. Rev. Lett.* **125**, 058103 (2020).

49. L. Wasserman, *All of Statistics: A Concise Course in Statistical Inference* (Springer Science & Business Media, 2013).

50. M. D. Keller, B. J. Norton, D. J. Farrar, P. Rutschman, M. Marvit, A. Makagon, Optical tracking and laser-induced mortality of insects during flight. *Sci. Rep.* **10**, 14795 (2020).

51. J. M. Patt, A. Makagon, B. Norton, M. Marvit, P. Rutschman, M. Neligeorge, J. Salesin, An optical system to detect, surveil, and kill flying insect vectors of human and crop pathogens. *Sci. Rep.* **14**, 8174 (2024).

52. E. R. Mullen, P. Rutschman, N. Pegram, J. M. Patt, J. J. Adamczyk, E. Johanson, Laser system for identification, tracking, and control of flying insects. *Opt. Express* **24**, 11828–11838 (2016).

53. J. S. Kennedy, "The visual responses of flying mosquitoes," in *Proceedings of the Zoological Society of London* (Zoological Society of London, 1940), vol. 109–4, pp. 221–242.

54. M. Geier, O. J. Bosch, J. Boeckh, Influence of odour plume structure on upwind flight of mosquitoes towards hosts. *J. Exp. Biol.* **202**, 1639–1648 (1999).

55. A. Cribellier, J. Spitzen, H. Fairbairn, C. van de Geer, J. L. van Leeuwen, F. T. Muijres, Lure, retain, and catch malaria mosquitoes. How heat and humidity improve odour-baited trap performance. *Malar. J.* **19**, 357 (2020).

56. B. A. Amos, S. A. Ritchie, R. T. Cardé, Attraction versus capture II: Efficiency of the BG-Sentinel trap under semifield conditions and characterizing response behaviors of male *Aedes aegypti* (Diptera: Culicidae). *J. Med. Entomol.* **57**, 1539–1549 (2020).

57. A. Hinze, J. Lantz, S. R. Hill, R. Ignell, Mosquito host seeking in 3D using a versatile climate-controlled wind tunnel system. *Front. Behav. Neurosci.* **15**, 643693 (2021).

58. A. W. A. Brown, Factors in the attractiveness of bodies for mosquitoes. *Nature* **167**, 202–202 (1951).

59. A. Frishman, P. Ronceray, Learning force fields from stochastic trajectories. *Phys. Rev. X* **10**, 021009 (2020).

60. P. Ronceray, Learning dynamical models from stochastic trajectories. arXiv:2406.02363 (2024).

61. D. B. Brückner, N. Arlt, A. Fink, P. Ronceray, J. O. Rädler, C. P. Broedersz, Learning the dynamics of cell–cell interactions in confined cell migration. *Proc. Natl. Acad. Sci. U.S.A.* **118**, e2016602118 (2021).

62. S. L. Brunton, J. L. Proctor, J. N. Kutz, Discovering governing equations from data by sparse identification of nonlinear dynamical systems. *Proc. Natl. Acad. Sci. U.S.A.* **113**, 3932–3937 (2016).

63. N. M. Mangan, J. N. Kutz, S. L. Brunton, J. L. Proctor, Model selection for dynamical systems via sparse regression and information criteria. *Proc. R. Soc. A. Math. Phys. Eng. Sci.* **473**, 20170009 (2017).

64. N. M. Mangan, T. Askham, S. L. Brunton, J. N. Kutz, J. L. Proctor, Model selection for hybrid dynamical systems via sparse regression. *Proc. R. Soc. A* **475**, 20180534 (2019).

65. A. A. Neath, J. E. Cavanaugh, The Bayesian information criterion: Background, derivation, and applications. *Wiley Interdiscip. Revi. Comput. Stat.* **4**, 199–203 (2012).

66. J. D. Pleil, M. A. G. Wallace, M. D. Davis, C. M. Matty, The physics of human breathing: Flow, timing, volume, and pressure parameters for normal, on-demand, and ventilator respiration. *J. Breath Res.* **15**, 042002 (2021).

67. A. J. Grant, R. J. O'Connell, Age-related changes in female mosquito carbon dioxide detection. *J. Med. Entomol.* **44**, 617–623 (2007).

68. F. Farrell, J. Tailleur, D. Marenduzzo, M. C. Marchetti, Pattern formation in self-propelled particles with density-dependent motility. *Phys. Rev. Lett.* **108**, 248101 (2012).

69. Mosquito Simulation Portal (2025); https://github.com/f-chenyi/MosquitoProject.

70. Y. Li, X. Su, G. Zhou, H. Zhang, S. Puthiyakunnon, S. Shuai, S. Cai, J. Gu, X. Zhou, G. Yan, X.-G. Chen, Comparative evaluation of the efficiency of the BG-Sentinel trap, CDC light trap

and Mosquito-oviposition trap for the surveillance of vector mosquitoes. *Parasit. Vectors* **9**, 446 (2016).

71. W. Huang, J. Rodrigues, E. Bilgo, J. R. Tormo, J. D. Challenger, C. De Cozar-Gallardo, I. Pérez-Victoria, F. Reyes, P. Castañeda-Casado, E. J. Gnambani, D. F. de Sales Hien, M. Konkobo, B. Urones, I. Coppens, A. Mendoza-Losana, L. Ballell, A. Diabate, T. S. Churcher, M. Jacobs-Lorena, *Delftia tsuruhatensis* TC1 symbiont suppresses malaria transmission by anopheline mosquitoes. *Science* **381**, 533–540 (2023).

72. J. D. Rivera-Duarte, I. J. May-Concha, R. Vargas-Abasolo, M. X. Martínez-Castaneira, M. E. Farfán-Beltrán, B. Mendoza-Garfias, A. L. Flores-Villegas, A. Córdoba-Aguilar, Rewiring the vehicle: *Trypanosoma cruzi* parasites alter the antennae of their triatomine hosts. *Ecol. Evol.* **15**, e71164 (2025).

73. C. Zuo, C. Fei, A. Cohen, S. Kim, R. Carde, J. Dunkel, D. Hu, Original data of "Predicting mosquito flight behavior using Bayesian dynamical systems learning," Zenodo (2025); https://zenodo.org/records/15277051.

74. C. Zuo, C. Fei, A. E. Cohen, S. Kim, R. T. Carde, Code for "Predicting mosquito flight behavior using Bayesian dynamical systems learning," Zenodo (2025); https://zenodo.org/records/17956325.

75. A. Chandel, N. A. De Beaubien, A. Ganguly, G. T. Meyerhof, A. A. Krumholz, J. Liu, V. L. Salgado, C. Montell, Thermal infrared directs host-seeking behaviour in *Aedes aegypti* mosquitoes. *Nature* **633**, 615–623 (2024).

76. G. Gibson, A behavioural test of the sensitivity of a nocturnal mosquito, *Anopheles gambiae*, to dim white, red and infra-red light. *Physiol. Entomol.* **20**, 224–228 (1995).

77. PFMD Key Specifications (2020); https://photonicsentry.com/uploads/PFMD_Sell_Sheet.pdf.

78. M. C. Göpfert, H. Briegel, D. Robert, Mosquito hearing: Sound-induced antennal vibrations in male and female *Aedes aegypti*. *J. Exp. Biol.* **202**, 2727–2738 (1999).

79. A. Krogh, J. Hertz, "A simple weight decay can improve generalization," in *Advances in Neural Information Processing Systems*, J. Moody, S. Hanson, R. Lippmann, Eds. (Morgan-Kaufmann, Burlington, MA, 1991), vol. 4, pp. 950–957.

80. D. J. MacKay, A practical Bayesian framework for backpropagation networks. *Neural Comput.* **4**, 448–472 (1992).

81. D. J. MacKay, Bayesian interpolation. *Neural Comput.* **4**, 415–447 (1992).

82. J. K. Ghosh, M. Delampady, T. Samanta, *An Introduction to Bayesian Analysis: Theory and Methods* (Springer, 2006), vol. 725.

83. C. Rackauckas, Q. Nie, DifferentialEquations.jl – A performant and feature-rich ecosystem for solving differential equations in julia. *J. Open Res. Softw.* **5**, 15 (2017).

84. J. M. Joiner, A. S. Branca, M. G. Banfield, C. H. Downs, G. M. Muzio, J. H. Borden, Three-dimensional evaluation of the responses of two species of flies (Diptera) to an indoor light trap. *J. Econ. Entomol.* **117**, 2591–2598 (2024).
